# Vitreous protein networks around ANG2 and VEGF in proliferative diabetic retinopathy and the differential effects of aflibercept versus bevacizumab pre-treatment

**DOI:** 10.1038/s41598-022-25216-z

**Published:** 2022-12-06

**Authors:** Ingeborg Klaassen, Peter Avery, Reinier O. Schlingemann, David H. W. Steel

**Affiliations:** 1 Ocular Angiogenesis Group, Department of Ophthalmology, Amsterdam UMC Location University of Amsterdam, Meibergdreef 9, Amsterdam, The Netherlands; 2 Amsterdam Cardiovascular Sciences, Microcirculation, Amsterdam, The Netherlands; 3 Amsterdam Neuroscience, Cellular & Molecular Mechanisms, Amsterdam, The Netherlands; 4 Bioscience Institute, Newcastle University, Newcastle Upon Tyne, UK; 5 Sunderland Eye Infirmary, Sunderland, UK; 6 Department of Ophthalmology, University of Lausanne, Jules-Gonin Eye Hospital, Fondation Asile Des Aveugles, Lausanne, Switzerland

**Keywords:** Biomarkers, Retinal diseases

## Abstract

Extracellular signalling proteins interact in networks rather than in isolation. In this context we investigated vitreous protein levels, including placental growth factor (PlGF), angiopoietin-2 (ANG2) and vascular endothelial growth factor (VEGF), in patients with proliferative diabetic retinopathy (PDR) with variable disease severities, and after anti-VEGF pre-treatment. Vitreous samples of 112 consecutive patients undergoing vitrectomy for PDR and of 52 non-diabetic patients with macular holes as controls were studied. A subset of the PDR patients were treated with either aflibercept (AFB, *n* = 25) or bevacizumab (BVZ)/ranibizumab (RZB) (*n* = 13), before surgery. Antibody-based analysis of 35 proteins (growth factors and cytokines) showed a significant increase in expression levels of 27 proteins in PDR patients as compared to controls. In network analysis of co-regulated proteins, a strong correlation in expression levels between VEGF, PlGF, MCP1 and ANG2 was found, mostly clustered around ANG2. In the AFB treatment group, concentrations of several proteins were decreased, including VEGFR1, whereas interleukin 6 and 8 were increased as compared to untreated PDR patients. The observed differences in vitreous protein levels between the different treatments and untreated PDR patients may underlie differences in clinical outcomes in patients with PDR.

## Introduction

In 2010 it was estimated that 10.2% of people with diabetes had sight threatening retinopathy, which amounts to about 28 million people worldwide, 17 million of whom have proliferative diabetic retinopathy (PDR)^1^. These numbers are expected to rise due to the increasing prevalence of diabetes, aging of the population and increasing life expectancy. PDR in particular is a major problem, with between 5 and 50% of patients requiring vitrectomy surgery for its complications depending on the stage of disease at presentation^2,3^. Anti-vascular endothelial growth factor (VEGF) agents have been a breakthrough in treatment of macular oedema and more recently positive results have been shown in patients with PDR^4,5^. However, not all patients with clinically important retinopathy respond optimally to anti-VEGF treatment^6,7^. Furthermore, VEGF suppression can stimulate a switch to a pro-fibrotic growth factor mix, with pre-retinal fibrosis and tractional retinal detachment as a result^8^. Indeed, other cytokines and growth factors are known to play a role in the pathogenesis of PDR, with placental growth factor (PlGF) being one of them^9^. Like VEGF, PlGF is a member of the VEGF family of growth factors, in addition to VEGF-A, B, C, D in humans. The three-dimensional structure of PlGF is strikingly similar to that of VEGF-A, although the two proteins only share 42% of their amino acid sequence identity^10^. All VEGFs function by binding to one or more of three cell surface-bound tyrosine kinase receptors called vascular endothelial growth factor receptors (VEGFRs1-3), causing them to dimerise and be activated through transphosphorylation^11^.

PlGF has been reported to be upregulated after anti-VEGF therapy^13^ and was postulated to work as a redundant inducer of neovascularisation^14^. In mouse models, anti-PlGF antibodies reduce neovascularisation and vascular leakage, and inhibition of PlGF signalling appears to be as effective as VEGF blockade^9,15^. In addition, activation of protective factors by inhibition of PlGF signalling may confer additional benefits, including increased survival of retinal cells (neuroprotection), decreased capillary degeneration and pericyte loss, preservation of the blood-retinal barrier (BRB), inhibition of inflammation (e.g. infiltration of macrophages and leukocytes), and inhibition of collagen deposition^14–16^.

High levels of PlGF have been measured in the vitreous humour^17–21^ and in retinal tissue^19,21–23^ of eyes with DR. However, its exact association with different stages of DR is unclear and its correlation to other cytokines, inflammatory markers and growth factors of importance in DR is undefined. These are important, both for a full understanding of the role of PlGF in DR but also of relevance in therapies. Aflibercept (Eylea; AFB) is constructed from a chimeric VEGFR1/VEGFR2-based decoy receptor fused to the Fc fragment of IgG1 (i.e., VEGFR1/VEGFR2-Fc). Therefore, unlike bevacizumab (Avastin; BVZ) and ranibizumab (Lucentis; RZB) which block VEGFA action only, AFB binds PlGF and VEGFB as well. Studies have reported conflicting effects of these agents on PlGF levels in vitreous humour, with some finding a reduction in PlGF levels after RZB^24^, some finding an increase after RZB^25^, and others no effect with BVZ and RZB^26^. In addition, an increase in systemic PlGF after AFB treatment has been reported^27^. AFB has also recently been shown to block the action of galectin-1^28,29^, a VEGF independent angiogenic factor of importance in DR^30^. There have also been noted to be differences in inflammatory markers and fibrosis after anti PlGF as compared to anti VEGF therapy in mouse models of neovascular retinal disease^9^, but the network interaction and effect of blocking these additional proteins in patients with PDR is unclear. There has been short term evidence of higher efficacy of AFB as compared to RZB and BVZ in patients treated for diabetic retinopathy which may be due to differences in these differentially effected protein networks after treatment^31,32^.

To clarify these protein network effects, we collected vitreous in a cohort of patients undergoing vitrectomy for the complications of diabetic retinopathy, and analysed the samples for a range of growth factors and cytokines. A proportion of patients were pre-treated with either BVZ/RZB or AFB as part of their pre-operative preparation for surgery and we investigated the effect of these agents on vitreous protein levels. In addition, a variety of pre-, intra- and post-operative variables were recorded and related to the results of the vitreous protein assays.

## Results

### Patient characteristics

In total 112 vitreous samples of PDR patients and 52 samples of control patients were analysed (Table 1). Of the PDR patients, 25 were treated preoperatively with AFB (PDR-AFB) and 13 with BVZ or RZB (PDR-BVZ/RZB), and 74 were untreated. Treated PDR patients were on average significantly younger than untreated PDR patients (45.6 for PDR-AFB and 48.2 for PDR-BVZ/RZB vs. 57.2 for untreated), and untreated PDR patients were on average significantly younger than control patients (57.2 vs. 69.2). The treatment groups did not differ in other parameters assessed.

**Table 1:** Patient characteristics.

Characteristic	PDR Patients untreated (N = 74)	PDR patients treatment A (N = 25)	PDR patients treatment B (N = 13)	Controls (N = 52)	*P* value PDR versus CON	*P* value PDR-A/B versus PDR-U	*P* value PDR-A versus PDR-B
Age, mean ± SD, years	57.2 ± 14.3	45.6 ± 15.4	48.2 ± 12.0	69.2 ± 6.5	** < 0.001**	** < 0.001**	0.600
**Gender**					** < 0.001**	0.500	0.730
Female (%)	32 (43)	12 (48)	7 (54)	41 (77)			
**Diabetes mellitus**						0.100	0.290
Type 1	31 (42)	16 (64)	6 (46)				
Type 2, with diet	4 (5)	0	1 (8)				
Type 2, with tabs	13 (18)	1 (4)	0				
Type 2, with insulin	26 (35)	8 (32)	6 (46)				
Duration of diabetes, mean ± SD, years	22.3 ± 11.3	21.9 ± 9.9	20.8 ± 6.9			0.700	0.710
HbA1c, mean ± SD, mmol/mol	77.2 ± 22.0	77.7 ± 20.5	85.9 ± 20.1			0.470	0.260
**Ophthalmic status**							
Pre-op visual acuity, mean ± SD, logMAR	1.3 ± 0.6	1.2 ± 0.6	1.3 ± 0.5			0.720	0.750
Post-op visual acuity, mean ± SD, logMAR	0.4 ± 0.3	0.4 ± 0.3	0.5 ± 0.6			0.950	0.560
Primary indication for vitrectomy						0.450	0.490
Vitreous haemorrhage	16 (64)	9 (69)					
Macular traction/traction detachment	9 (36)	4 (31)					
**Neovascularisation activity**						** < 0.001**	0.480
No neovascularisation (0)	7 (9)	0	1 (8)				
Quiescent neovascularisation (1)	38 (51)	2 (8)	1 (8)				
Active neovascularisation (2)	29 (38)	23 (88)	11 (85)				
**Degree of vitreous haemorrhage**						0.160	0.900
No haemorrhage (Grade 0)	12 (16)	2 (8)	0				
Grade 1	23 (31)	12 (48)	7 (54)				
Grade 2	36 (47)	10 (40)	6 (46)				
No fundal view (Grade 3)	3 (4)	1 (4)	0				
**Degree of fibrosis**						**0.003**	0.850
No fibrosis (0)	16 (21)	0	0				
Grade 1	20 (27)	7 (28)	4 (31)				
Grade 2	25 (32)	8 (32)	3 (23)				
Abundant fibrous membranes (Grade 3)	13 (17)	10 (40)	6 (46)				
New vessel extent in disc areas. Mean, SD	4.6(4.4)	8.8(6.1)	8.9(7.8)			** < 0.001**	0.960
**New vessel location**						0.280	0.500
None (0)	18 (24)	4 (16)	1 (8)				
Disc only (1)	21 (28)	7 (28)	1 (8)				
Disc and posterior pole (2)	20 (27)	6 (24)	5 (38)				
Posterior pole and 1 quadrant aneriorly (3)	15 (20)	6 (24)	3 (23)				
Posterior pole and 2 or more quadrants anteriorly (4)	0	2 (8)	3 (23)				

All values given as number (percentage) unless stated.

*PDR* Proliferative diabetic retinopathy, *PVR* Proliferative vitreoretinopathy, *PDR-U* Untreated PDR patients, *PDR-A* PDR patients pre-operative treated with aflibercept, *PDR-B* PDR patients pre-operative treated with bevacizumab or ranibizumab, *SD* Standard deviation. *P* values < 0.05 are given, *P* values > 0.05 are indicated as "-". *P* values < 0.01 are considered to be statistically significant and are indicated in bold.

The control group contained more females than the untreated PDR group, but males and females were equally distributed in the two treated PDR groups. To further explore the influence of age and gender differences between groups, we log transformed all variables without zeros and fitted control versus PDR with gender as a factor and age as a covariate. We found that gender was never a significant factor and age only marginally significant in a few cases, whereas control versus PDR was highly significant for almost all the variables, with high R squared values in most cases (supplemental Table 1). These results strongly suggest that age and gender did not confound the protein levels tested between the groups.

As expected, the two anti-VEGF treated PDR groups showed some differences with the untreated PDR group, as the clinical decision to treat pre-operatively was determined by clinical signs such as severity of fibrovascular proliferation. Since in general the patients with the highest degree of neovascularization were treated preoperatively with anti-VEGF compounds through selection bias, expectedly the extent and activity of neovascularization was higher in the anti-VEGF treated PDR groups as compared to the untreated PDR group, and the degree of fibrosis was less. No significant differences were present in any of the variables between both anti-VEGF treated PDR groups (Table 1).

### Quantification of protein concentrations and activity in the control versus PDR eyes

The concentration of 33 proteins was quantified in the vitreous samples by means of Quantibody® arrays (Table 2). 26 proteins showed higher concentrations in vitreous of untreated PDR versus control patients, when using a Bonferroni corrected *p* value of 0.001 or less as statistically significant, of which 12 of the proteins by more than fivefold. The highest differences in protein levels were found for VEGF (98-fold) and PlGF (75-fold), followed by MMP9 (58-fold), IL-8 (30-fold,), adiponectin (18-fold), thrombospondin-1 (14-fold), angiopoietin-2 (13-fold), IL-1β (12-fold), IL-6 (tenfold), IGFBP1 (eightfold), ICAM-1 (sevenfold) and galectin-3 (5.5-fold). However, concentrations of some proteins were below the level of detection in the control group (MMP9, IL-8, and thrombospondin-1) or in both control and PDR groups (IL-1β), making these fold changes less reliable. In addition, for Betacellulin, GDNF, IL-10, PDGF-BB and Ubiquitin+1, the fold change in protein concentrations between the control group and the PDR group could not be determined, since the median protein concentration in the control group was zero. The most abundant proteins found in vitreous were adiponectin, VEGFR1 and tissue-inhibitor of metalloproteases-1 (TIMP-1). Considering a *P* value < 0.05, MMP2 levels were lower in PDR patients (*P* = 0.0058) as compared to controls (Table 2). IGF1 could not be detected in any of the control or PDR samples. Distribution of individual data points is shown in Figs. 3 and 4. The maximum VEGFA concentration in control samples was 403.1 pg/ml and 92% of the PDR samples had higher concentrations; The maximum concentration of PlGF was 41 pg/ml in control samples and 95% of the PDR samples had higher concentrations.

**Table 2:** Protein concentrations in vitreous of control and PDR patients.

Protein	LOD	CON (n = 52)		PDR (n = 74)		Fold change	*P* value
		Median	Q1–Q3	Median	Q1–Q3		
**Array**							
Adiponectin	860	4879	873–19,565	85,703	44,408–222,093	**17.57**	** < 0.0001**
Angiopoietin-1	3.4	7.0	*2.1*–8.6	9.1	3.9–15.6	1.30	0.0033
Angiopoietin-2	157	437	217–640	5761	2425–13,256	**13.17**	** < 0.0001**
Betacellulin	75.9	*0.0*	*0.0*–*0.1*	*22.5*	*0.0*–*64.6*	+	< 0.0001
Galectin-1	41.7	215	141–311	778	461–1127	3.61	< 0.0001
Galectin-3	3.0	6.2	3.4–10.1	33.7	16.8–48.6	**5.45**	** < 0.0001**
GDF-15	9.2	1784	994–3487	5947	4679–8055	3.33	< 0.0001
GDNF	30.8	*0.0*	*0.0*–*3.4*	*12.4*	*0.0*–43.5	+	< 0.0001
HGF	5.9	279	204–447	626	403–1075	2.24	< 0.0001
ICAM-1	2.2	19.6	8.9–50.8	135.5	103.2–196.5	**6.92**	** < 0.0001**
IGF-1	591	*0.0*	*0.0*–*0.0*	*0.0*	*0.0*–*0.0*		
IGFBP-1	13.1	247	84.6–649	2057	637–5945	**8.34**	** < 0.0001**
IGFBP-3	161	2734	1749–4317	8994	5965–14,442	3.29	< 0.0001
IL-1β	24.8	*0.7*	*0.0*–*6.5*	*8.6*	*0.4*–*19.8*	**11.90**	** < 0.0001**
IL-6	38.5	50.2	*6.7*–91.8	485	322–861	**9.66**	** < 0.0001**
IL-8	73.2	*27.2*	*9.3*–*72.0*	811	521–1425	**29.87**	** < 0.0001**
IL-10	21.9	*0.0*	*0.0*–*2.8*	*9.7*	*4.2*–*17.0*	+	< 0.0001
MCP-1	21.4	2533	2278–3095	4023	3385–4637	1.59	< 0.0001
MMP-2	8.7	33.5	15.7–53.8	17.5	9.8–40.0	− 1.91	0.0058
MMP-9	5.4	*0.7*	*0.0*–5.8	42.1	12.7–74.1	**57.83**	** < 0.0001**
NOV	25.2	4245	2878–5914	5012	3944–7399	1.18	0.0346
NRG1-β1	3.4	*1.9*	*0.3*–3.5	*2.2*	*1.1*–4.0	1.16	0.3177
PDGF-AA	209	233	*0.0*–513	728	346–1712	3.13	< 0.0001
PDGF-BB	1.2	*0.0*	*0.0*–*0.0*	*0.2*	*0.0*–*1.0*	+	< 0.0001
PIGF	12.1	*5.5*	*0.5*–*11.4*	412	124–848	**74.95**	** < 0.0001**
TGFβ2	7.1	178	107–259	167	63.5–304	0.94	0.5925
Thrombospondin-1	23.0	*9.4*	*0.3*–*22.5*	131.9	46.0–372.1	**14.02**	** < 0.0001**
TIMP-1	881	100,300	87,660–124,151	137,260	125,627–152,430	1.37	< 0.0001
TNFα	40.5	*7.8*	*0.0*–*27.3*	*27.4*	*17.0*–42.6	3.53	< 0.0001
Ubiquitin+1	62.2	*0.0*	*0.0*–*0.0*	*0.0*	*0.0*–*43.0*	+	0.0066
VEGF R1	297	79,651	62,727–103,456	127,034	108,894–168,201	1.59	< 0.0001
VEGF R2	153	4101	3072–5990	6958	4829–9715	1.70	< 0.0001
VEGFA	15.0	107	73.0–152	10,425	1652–36,345	**97.84**	** < 0.0001**
**ELISA**							
CTGF	5.0	77,306	35,025–141,128	337,009	214,750–705,958	4.36	< 0.0001
IGFBP3	80.0	1824	1035–4101	7421	5071–9999	4.07	< 0.0001
PEDF	0.6	27.1	15.8–68.4	11.5	7.8–17.9	− 2.35	< 0.0001
PlGF	7.0	*0.0*	0.0–2.9	96.0	25.8–211	+	** < 0.0001**
VEGF	5.0	2.3	0.0–32.9	730	87.8–2836	**317**	** < 0.0001**
**Zymography**							
MMP2	−	1,027,790	0.0–2,467,665	1,252,856	0.0–2,737,807	1.22	0.8558
Pro-MMP2	−	4,184,190	1,519,850–7,270,280	6,025,338	3,639,811–8,441,355	1.44	0.0032
Pro-MMP9	−	0.0	0.0–812,512	1,333,870	0.0–3,463,083	+	0.1057

PEDF data is presented in µg/ml, Zymography data is expressed as intensity of bands in arbitrary units. All other protein data are presented in median pg/mL with 1st and 3rd quartile values. BDL, below limit of detection (LOD). Levels that are lower than LOD are underlined and indicated in italics, and should be considered less reliable. Fold changes with '+' present values in PDR that are higher as compared to controls with zero median levels. The Mann–Whitney U test was used to assess statistical differences between PDR and control patients. Fold changes higher than 5-fold and a P-value < 0.001 are indicated in bold.

As a way to validate and confirm the array results, VEGFA, PlGF and IGFBP3 were also measured by ELISA (Table 2 and Fig. 2), which also allows more accurate quantification. For ELISA data a Bonferroni corrected *p* value of 0.01 (0.05/5) or less was used. Whereas the fold change of IGFBP3 was comparable to that in the array, the fold change of VEGFA was much higher, and that of PlGF could not be determined due to a median zero value in the control group. However, using Bland Altman plots^33^, we found that both methods were in broad agreement with each other for all three proteins (Supplemental Fig. 1).

PEDF and CTGF levels were measured separately by ELISA (Table 2), because the antibodies could not be combined with others on the arrays due to potential cross-reactivity. Significantly lower protein concentrations for PEDF were found (2.4-fold), whereas concentrations for CTGF were higher (4.4-fold). Protein activity of pro-MMP9, pro-MMP2 and MMP2 was determined by zymography (Table 2). For zymography data, a Bonferroni corrected *P* value of 0.0167 (0.05/3) was used. Higher activity of pro-MMP9 (*P* = 0.0131) and pro-MMP2 (*P* = 0.0085) was found in PDR patients as compared to controls, whereas MMP2 activity was similar.

In summary, 27 of the 35 proteins analysed showed increased levels in PDR patients compared to controls. Of these proteins, 12 showed increases of more than fivefold, with the highest levels for VEGF and PlGF. Protein levels of PEDF were significantly lower in PDR patients compared to controls.

### Co-regulation of proteins

**Figure 1:**
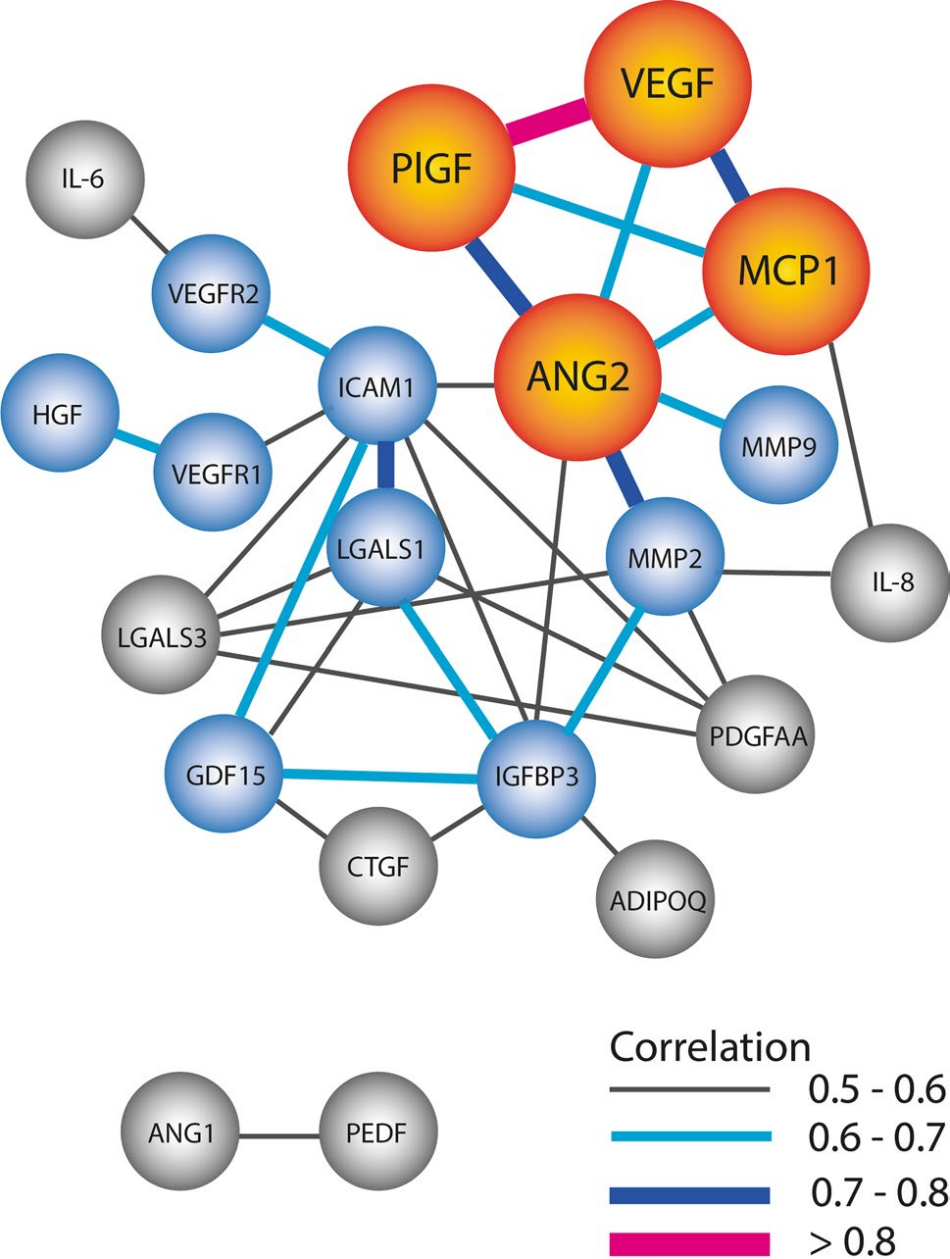
Co-regulation of proteins. Protein concentrations in the untreated PDR group were analysed using Pearson’s regression. This forms a network of proteins. Only correlations and proteins that had a correlation coefficient higher than 0.4 and *P* value < 0.01 are shown. Orange indicates the proteins that had the strongest correlation with other proteins; blue circles had at least one correlation > 0.6; grey: only moderate correlations. Line thickness indicates the strength of the correlation, with correlation coefficients between 0.4 and 0.5 being moderate and > 0.6 strong.

When using data from the untreated PDR group, Spearman’s rank correlation analysis revealed that a cluster of mainly four proteins had strong correlations among samples: VEGF, PlGF, angiopoietin 2 (ANG2) and monocyte chemoattractant protein 1 (MCP1) (Fig. 1). For this purpose, protein data from arrays were used for all proteins except for CTGF and PEDF, which were derived from ELISA data. Protein data from ELISA’s for VEGF, PlGF, and IGFBP3 gave similar results to those from arrays. VEGF and PlGF concentrations had the highest correlation among all samples (Spearman’s *r* = 0.840, *P* < 0.0001). Correlations within this quartet were also high for ANG2 and PlGF (*r* = 0.712), ANG2 and VEGF (*r* = 0.640), VEGF and MCP1 (*r* = 0.726), PlGF and MCP1 (*r* = 0.615) and ANG2 and MCP1 (*r* = 0.614), all with P < 0.0001. In addition, strong correlations were found between ANG2 and MMP2 (*r* = 0.777), MMP9 (*r* = 0.653) and galectin 1 (LGALS1) (*r* = 0.624) and between intercellular adhesion molecule 1 (ICAM1) and LGALS1 (*r* = 0.706), all with *P* < 0.0001. Additional strong and moderate correlations were found between proteins and indicated in Fig. 1, all correlations can be found in Supplemental Table 2. In this correlative network of proteins, most relations centred around ANG2. Figure 1 shows that VEGF had correlations with 3 proteins, while ANG2 had correlations with 7 proteins. Moreover, the proteins with which ANG2 was correlated also appeared to have significant correlations with other proteins and these with each other. This suggests a network of markedly co-regulated proteins around ANG2.

When comparing the zymography data with the array data, a correlation was found for MMP9 protein levels and ProMMP9 activity (*r* = 0.365, *P* = 0.018) and ProMMP9 activity with ProMMP2 activity (*r* = 0.445, *P* = 0.011), but not for MMP2 protein levels and MMP2 activity or ProMMP2 activity and not for MMP2 activity versus ProMMP2 activity (Supplemental Fig. 2).

These results showed that there was a strong correlation in expression levels of VEGF, PlGF, MCP1 and ANG2. In this network of co-regulated proteins, most of the correlating proteins were clustered around ANG2.

### Relationship and correlation analysis of vitreous protein concentrations and clinical features of PDR

By statistical analysis using Spearman’s correlation test, we found relationships with clinical features as defined in Table 1 and protein levels in untreated PDR patients (Table 3). Pre-operative visual acuity was related to levels of IGFBP3, MCP1 and platelet-derived growth factor (PDGF)-BB. The protein concentrations of these three proteins were also correlated with each other. There were no significant correlations with HbA1c or post-operative visual acuity.

**Table 3:** Correlations between protein levels and visual acuity.

		PRE-OP VA	IGFBP3-ELISA	MCP-1
IGFBP3-ELISA	R	0.318		
	P	**0.009**		
MCP-1	R	0.344	0.320	
	P	**0.003**	**0.008**	
PDGF-BB	R	0.322	0.384	0.222
	P	**0.006**	**0.001**	0.057

Spearman's correlation coefficents (R) and *P* values (*P*) are given. Significant differences (*P* < 0.01) are indicated in bold.

The Kruskal–Wallis test was used to test for significant differences in median protein levels between groups of defined clinical features (Table 4). Two groups, one where the primary indication for surgery was vitreous haemorrhage, and the other for macular traction/traction detachment, were tested. Two proteins, namely IGFBP1 and PDGF-BB levels were significantly higher in the vitreous haemorrhage group. In a similar way, two groups were generated for other clinical features. The degree of vitreous haemorrhage was divided in two groups: degree 0 + 1 versus 2 + 3. The protein levels of IGFBP3, MMP9 and PDGF-BB were significantly higher in the denser haemorrhage group. For the activity of neovascularisation, one group with no or quiescent neovascularisation (0 + 1) and another group with active neovascularisation (2) were tested. VEGF and PlGF levels (from ELISA and array data) and MCP1 levels were significantly higher in the group with active neovascularisation. New vessel extent was divided in three groups: 0 + 1 versus 2 versus 3 + 4 (as defined in Table 1). The levels of ANG1 were significantly lower in the first group as compared with the other two groups (*P* = 0.001). The degree of fibrosis was divided in two groups: degree 0 + 1 versus 2 + 3. MMP9 levels were significantly higher in the second group as compared to the first group (*P* < 0.0001). There was no significant association between any of the tested proteins and postoperative vitreous cavity haemorrhage, nor the need for revision surgery for recurrent tractional complications.

**Table 4:** Differences in clinical features between defined groups of untreated PDR patients.

Variable	Contrast	Protein	Median protein levels (pg/ml)			*P* value
			Group 1	Group 2	Group 3	
**Primary indication for vitrectomy**	VH versus VMT/TRD					
		IGFBP1	2815	681		0.009
		PDGF-BB	0.361	0.000		0.007
**Neovascularisation activity**	0 + 1 versus 2					
		VEGF-ELISA	465	3010		< 0.001
		VEGFA	5250	29,006		< 0.001
		PlGF-ELISA	43.51	219.71		< 0.001
		PlGF	190	855		< 0.001
		MCP-1	3770	4403		0.003
**Degree of haemorrhage**	0 + 1 versus 2 + 3					
		CTGF-ELISA	255,755	521,370		0.003
		IGFBP3-ELISA	6293	8121		0.005
		HGF	490	754		0.001
		MCP-1	3494	4276		0.002
		MMP-9	28.86	51.81		0.005
		PDGF-BB	0.000	0.514		< 0.001
	0 + 1 versus 2 + 3					
		MMP-9	23.63	64.99		< 0.001
**Diabetes type**	Type 1 versus 2					
		MCP-1	3494	4182		0.003
		PlGF	189	531		0.004
		VEGFA	4540	20,936		0.004
		GDF-15	5213	6944		0.002
**New vessel location**	0 + 1 versus 2 versus 3 + 4					
		ANGPT1	3.50	9.03	13.50	0.001

The null hypothesis that all medians are equal was analysed using the Kruskal–Wallis test. *P* values represent the significance of differences between group medians. Significant differences (*P* < 0.01) are given. Variables are defined in Table 1.

*VH* Vitreal haemorrhage, *VMT* Vitreo-macular traction, *TRD* Traction retinal detachment.

Together, these results showed that IGFBP3, MCP1 and PDGF-BB were related to pre-operative visual acuity. IGFBP1 and PDGF-BB were more related with vitreous haemorrhage than with macular traction/detachment. IGFBP3, MMP9 and PDGF-BB were related to the degree of haemorrhage, VEGF, PlGF and MCP-1 to the degree of neovascularisation, ANG1 to the degree of vessel extent, and MMP9 to the degree of fibrosis.

### Effect of pre-operative anti-VEGF treatment on protein concentrations

**Figure 2:**
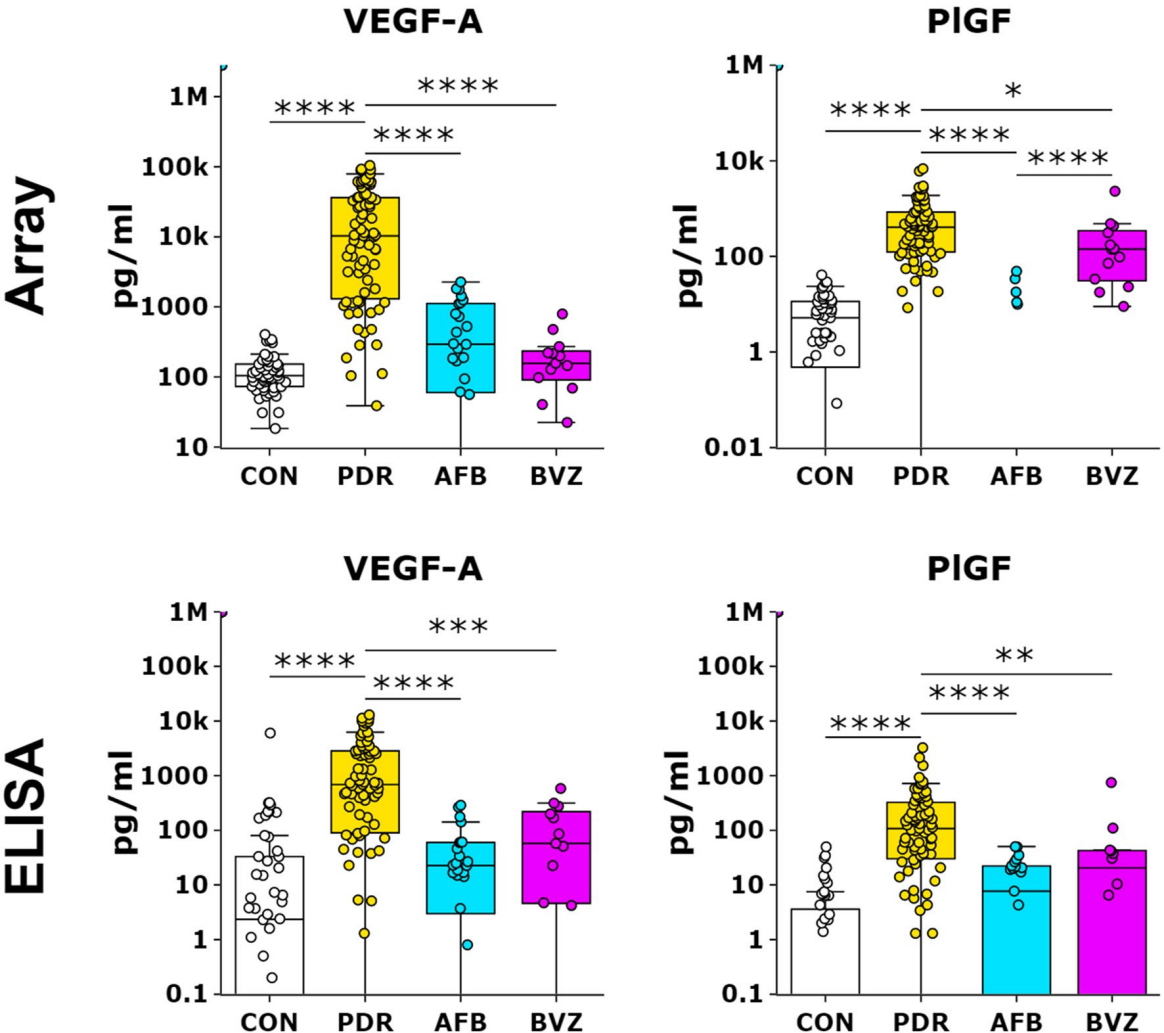
Effect of pre-operative anti-VEGF treatment on VEGFA and PlGF protein concentrations. Box plots indicate median, min and max and 1st and 3rd quartile. Protein concentrations for controls, untreated PDR patients (PDR), PDR patients treated with AFB (AFB) and PDR patients treated with BVZ or RZB (BVZ) are presented as pg/ml in log10 scale. Graphs in the upper row were obtained from array data, graphs in the lower row from ELISA data. *P < 0.05; **P < 0.01; ***P < 0.001; ****P < 0.0001.

Prior to vitrectomy, 13 patients received BVZ (*n* = 10) or RZB (*n* = 3) (grouped as PDR-BVZ/RZB) and 25 patients received AFB (PDR-AFB). Protein concentrations and results of the zymography assays were compared between the PDR-AFB, PDR-BVZ/RZB group and untreated PDR (PDR-U) group (Table 5). In the protein array analysis, VEGF concentrations were much lower in both treatment groups as compared to the untreated PDR group (PDR-AFB, 36-fold, *P* < 0.0001; PDR-BVZ/RZB, 67-fold, *P* < 0.0001), with no difference between the two treatment groups (Table 5 and Fig. 2). After AFB treatment, median PlGF concentrations were zero, whereas in the PDR-BVZ/RZB group median concentrations were 141.7 pg/ml, but still 12-fold lower as compared to the PDR-U group (*P* = 0.02) (Table 5 and Fig. 2). Similar results were found with the ELISA’s for VEGF and PlGF (Table 5 and Fig. 2). No statistically significant differences in protein levels were found between patients treated with BVZ and RZB.

**Table 5:** Protein concentrations in vitreous of PDR patients that were pre-operatively treated with anti-VEGF.

Protein	LOD	PDR-A (n = 25)		PDR-B (n = 13)		PDR-A versus PDR-U		PDR-B versus PDR-U		PDR-B versus PDR-A	
		Median	1st Q–3rd Q	Median	1st Q–3rd Q	FC	*P* value	FC	*P* value	FC	*P* value
**Array**											
Adiponectin	860	167,824	72,596–278,865	66,265	54,210–138,247	2.0	0.177	− 1.3	0.333	− 2.5	0.011
Angiopoietin-1	3.4	17.8	7.6–34.3	9.7	5.7–16.8	2.0	0.026	1.1	0.963	− 1.8	0.018
Angiopoietin-2	157	14,115	2467–22,369	4940	2628–14,132	2.5	0.322	− 1.2	0.807	− 2.9	0.173
Betacellulin	75.9	*0.0*	*0.0*–*0.0*	*3.9*	*0.0*–*54.1*	*–*	0.0013	− 5.7	0.848	+	0.555
Galectin-1	41.7	692	305–898	506	408–629	− 1.1	0.083	− 1.5	0.143	− 1.4	0.658
Galectin-3	3.0	49.5	31.6–56.7	22.0	11.4–47.5	1.5	0.027	− 1.5	0.229	− 2.2	0.008
GDF-15	9.2	6363	5330–7245	5838	4342–7071	1.1	0.912	1.0	0.470	− 1.1	0.547
GDNF	30.8	*0.0*	*0.0*–0.0	*0.0*	0.0–5.8	–	< 0.0001	–	0.023	–	0.353
HGF	5.9	973	354–1386	501	346–906	1.6	0.231	− 1.2	0.585	− 1.9	0.169
ICAM-1	2.2	187	127–224	127	96.7–152	1.4	0.066	− 1.1	0.389	− 1.5	0.020
IGF-1	591	BDL	–	BDL	–						
IGFBP-1	13.1	3992	1369–6147	982	766–1593	1.9	0.326	− 2.1	0.113	− 4.1	0.009
IGFBP-3	161	9729	5135–16,178	6281	5388–9282	1.1	0.856	− 1.4	0.058	− 1.5	0.028
IL-1β	24.8	*0.0*	*0.0*–*0.0*	*1.9*	*0.0*–*7.5*	−	< 0.0001	− 4.4	0.172	+	0.173
IL-6	38.5	1456	595–3478	1119	762–1482	3.0	0.002	2.3	0.007	− 1.3	0.091
IL-8	73.2	2095	1247–3004	1587	935–2000	2.6	0.0004	2.0	0.043	− 1.3	0.318
IL-10	21.9	*0.0*	*0.0*–*3.8*	*10.5*	*5.3*–*16.7*	−	< 0.0001	1.1	0.814	+	0.908
MCP-1	21.4	3375	3194–3762	3318	3112–3815	− 1.2	0.002	− 1.2	0.043	1.0	0.598
MMP-2	8.7	15.7	*0.0*–40.1	19.9	9.7–38.3	− 1.1	0.298	1.1	0.667	1.3	0.268
MMP-9	5.4	85.3	*2.9*–110	50.6	27.0–79.9	2.0	0.257	1.2	0.278	− 1.7	0.896
NOV	25.2	6107	4600–8703	4866	3261–5622	1.2	0.131	1.0	0.321	− 1.3	0.183
NRG1-β1	3.4	0.0	0.0–0.0	*0.1*	*0.0*–2.7	–	< 0.0001	− 18.0	0.060	+	0.277
PDGF-AA	209	*0.0*	*0.0–*882	778	*0.0*–2649	–	0.0003	1.1	0.428	+	0.536
PDGF-BB	1.2	*0.2*	*0.0–1.1*	*0.0*	*0.0*–*0.3*	1.1	0.522	–	0.383	− 45.65	0.523
PIGF	12.1	*0.0*	*0.0–0.0*	141.7	33.5–314.2	−	< 0.0001	− 2.9	0.020	+	0.083
TGFβ2	7.1	144	74.2*–*489	125	65.5–181	− 1.2	0.950	− 1.3	0.463	− 1.2	0.147
Thrombospondin-1	23.0	157	*0.0–*780	186	89.9–466	1.2	0.414	1.4	0.881	1.2	0.563
TIMP-1	881	150,417	131,312–171,530	136,308	128,425–153,733	1.1	0.078	1.0	0.576	− 1.1	0.822
TNFα	40.5	*0.0*	*0.0*–*3.4*	*20.0*	*12.0*–48.3	–	< 0.0001	− 1.4	0.640	+	0.011
Ubiquitin+1	62.2	*0.0*	*0.0*–*25.7*	*0.0*	*0.0*–*57.2*	–	0.888	–	0.414	–	0.571
VEGFR1	297	10,074	7550–11,525	113,522	90,772–168,454	**− 12.6**	**< 0.0001**	− 1.1	0.407	**11.3**	** < 0.0001**
VEGFR2	153	8180	5824–10,156	6498	5157–8780	1.2	0.442	− 1.1	0.763	− 1.3	0.209
VEGFA	15.0	292	60.8–1108	157	97.5–221	**− 35.7**	** < 0.0001**	**− 66.5**	** < 0.0001**	− 1.9	0.015
**ELISA**											
CTGF	5.0	549,892	266,993–836,699	287,454	165,074–689,946.9	1.6	0.213	0.9	0.660	− 1.1	0.259
IGFBP3	80.0	7977	5706–11,312	7183	5957–9105	1.1	0.433	1.0	0.847	− 1.1	0.119
PEDF	0.6	13.4	9.0–18.3	13.7	12.4–17.2	1.2	0.553	1.2	0.325	1.0	0.265
PlGF	7.0	7.7	*0.0*–22.1	20.5	*0.0*–42.2	**− 12.5**	** < 0.0001**	− 4.7	0.008	2.7	0.895
VEGF	5.0	22.6	*3.7*–59.5	57.5	*4.7*–200	**− 32.3**	** < 0.0001**	**− 12.7**	**0.0004**	2.5	0.243
**Zymography**											
MMP2	–	1,682,720	0.0–3,363,033	2,039,355	0.0–3,514,698	1.3	0.314	1.6	0.554	1.2	0.549
Pro-MMP2	–	6,184,134	2,884,669–9,130,790	7,272,447	3,967,305–10,904,740	1.0	0.910	1.2	0.676	1.2	0.556
Pro-MMP9	–	4,050,770	995,326–7,038,088	2,330,477	860,941–3,173,841	3.0	0.009	1.7	0.215	− 1.7	0.606

PEDF data is presented in µg/ml, Zymography data is expressed as intensity of bands in arbitrary units. All other protein data are presented in median pg/mL with 1st and 3rd quartile values. BDL, below limit of detection (LOD); FC, fold change; PDR-A, PDR patients treated with aflibercept; PDR-B, PDR patients treated with bevacizumab or ranubizumab. Values that are based on protein levels that are lower than LOD are underlined and indicated in italics, and should be considered less reliable. Fold changes with '+' or '-' present higher(+) or lower(-) protein levels which could not be determined because of zero median levels in one or more groups. The Mann-Whitney U test was used to assess statistical differences between both treatment groups. Fold changes higher than 5-fold and significant differences (P < 0.001) are indicated in bold.

**Figure 3:**
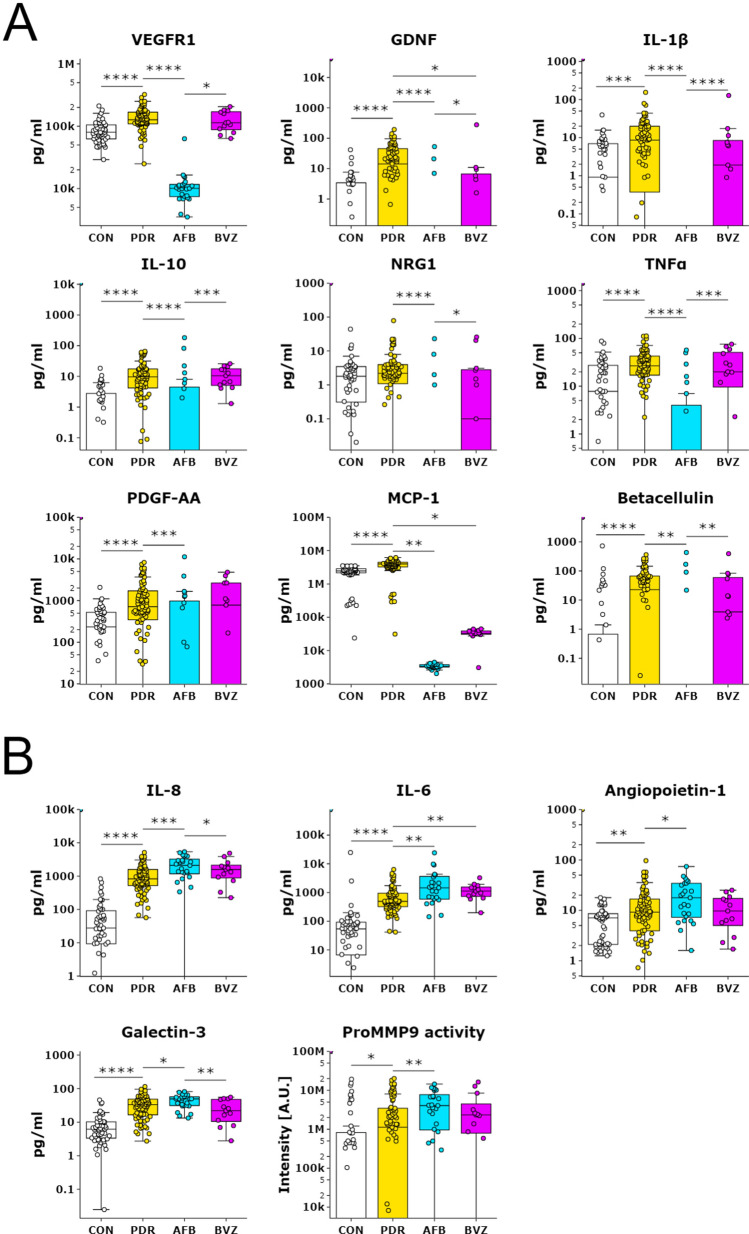
Effect of pre-operative anti-VEGF treatment on protein concentrations. Box plots indicate median, min and max and 1st and 3rd quartile. Protein concentrations for controls, untreated PDR patients (PDR), PDR patients treated with AFB (AFB) and PDR patients treated with BVZ or RZB (BVZ) are presented as pg/ml in log10 scale. (**A**) Proteins that showed lower levels in the PDR-AFB group as compared to the untreated PDR group. (**B**) Proteins that showed higher levels in the PDR-AFB group as compared to the untreated PDR group. In addition, some proteins showed differences in protein levels between PDR-BVZ/RZB and untreated PDR and/or PDR-AFB groups. ProMMP9 activity is expressed as intensity of bands in arbitrary units. *P < 0.05; **P < 0.01; ***P < 0.001; ****P < 0.0001.

**Figure 4:**
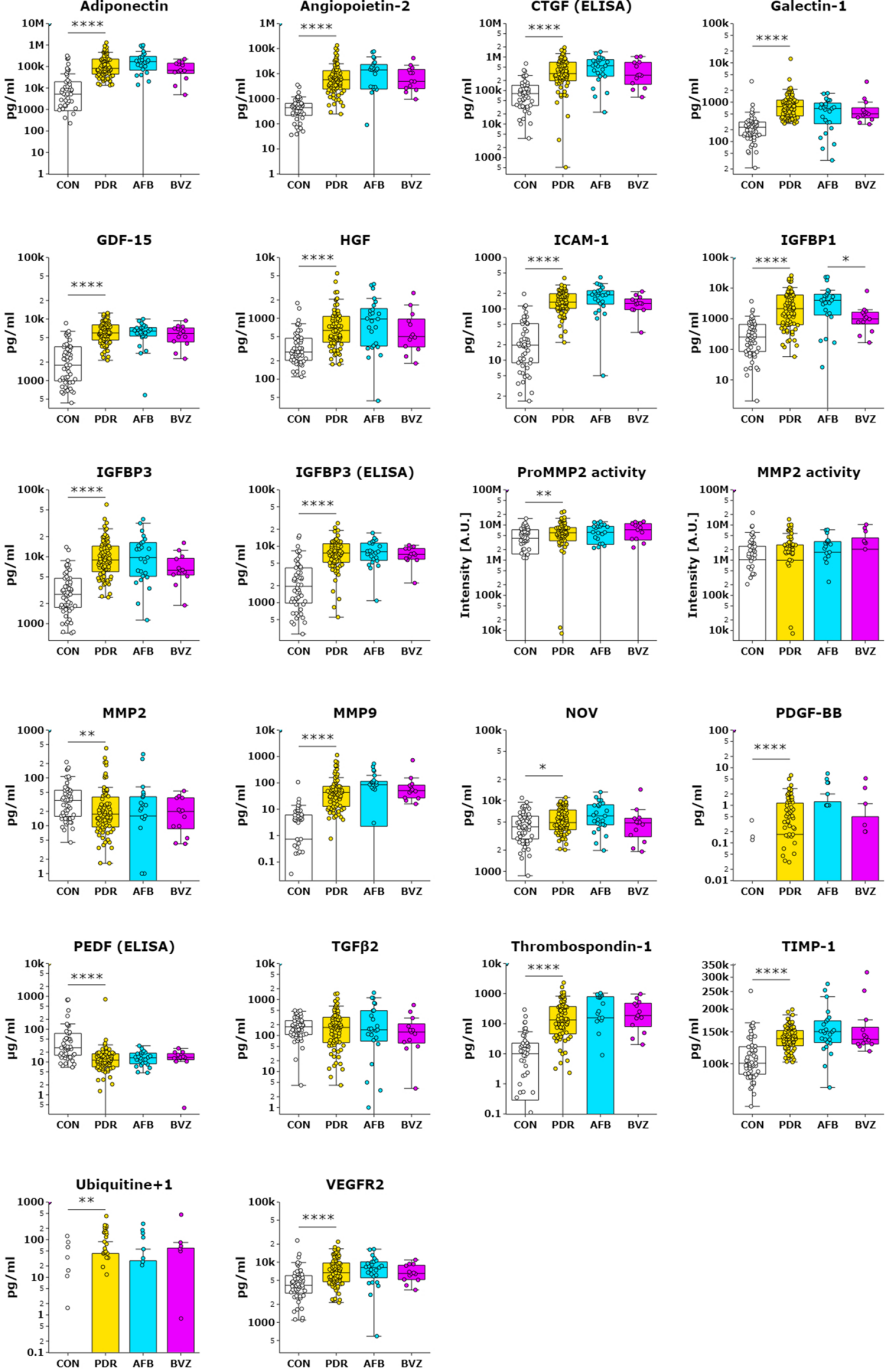
Proteins for which no difference in protein levels was found after anti-VEGF treatment. Box plots indicate median, min and max and 1st and 3rd quartile. Protein concentrations for controls, untreated PDR patients (PDR), PDR patients treated with AFB (AFB) and PDR patients treated with BVZ or RZB (BVZ) are presented in log10 scale. Two proteins are shown as data obtained by ELISA (IGFBP3 and PEDF), activity of ProMMP2 and MMP2 was determined by zymography, all other data was obtained from array analysis. *P < 0.05; **P < 0.01; ***P < 0.001; ****P < 0.0001.

A striking reduction in VEGFR1 (FLT1) protein levels was observed in the AFB treatment group, at 13-fold lower median levels as compared to untreated PDR group and 11-fold lower as compared to the BVZ/RZB treatment group (Table 5 and Fig. 3). Furthermore, after AFB treatment, glial cell line-derived neurotrophic factor (GDNF), interleukin (IL)-1β, IL-10, neuregulin 1 (NRG1), tumor necrosis factor (TNF)α and PDGF-AA were also lower (median protein level of zero) than in the PDR-U group (when using a Bonferroni-adjusted *P* value of 0.001 or less, Fig. 3A). Of these proteins, IL-1β, IL-10 and TNFα levels were also significantly lower in the PDR-AFB group as compared to the PDR-BVZ/RZB group. Considering a less stringent *P* value of 0.05, protein levels of MCP-1 and Betacellulin were lower in the PDR-AFB group (Fig. 3A), and MCP-1 and GDNF were lower in the PDR-BVZ/RZB group as compared to the PDR-U group (*P* < 0.05). Again, since most protein levels were below the level of detection, caution must be taken with these results which may be less reliable (Table 5). IL-8, IL-6 (both with *P* < 0.001), Angiopoietin-1 and Galectin-3 (both with *P* < 0.05) showed higher median levels in the PDR-AFB group (Fig. 3B). In addition, the median activity of Pro-MMP9 was higher in the PDR-AFB group as compared to the PDR-U group (Fig. 3B). All other protein levels in both treatment groups, including those of PEDF and CTGF, were not different as compared to the untreated PDR group (Fig. 4).

These results indicate that in addition to inhibiting their known targets VEGF and/or PlGF, the various anti-VEGF treatments also affected the levels of other proteins.

## Discussion

In this prospective study 164 vitreous samples of patients undergoing vitrectomy for the complications of PDR (*n* = 112) and for macular holes as controls (*n* = 52) were analysed by high-throughput protein screening. Some of the PDR patients were pre-operatively treated with different kinds of anti-VEGF agents (*n* = 38), which gave us the opportunity to compare protein levels between treated and untreated patients, and between the different anti-VEGF treatments. The present study is much larger than our previous similar study in vitreous of 7 control patients and of 13 PDR patients^34^. However, in general the results confirm the data of our previous study. Our results are also in accordance with previously published studies (reviewed by Kaštelan et al.^35^ and Mason et al.^36^), but we present several novel findings.

VEGF and PlGF showed the highest and most consistent increase in concentration in the vitreous of PDR patients as compared to controls (Table 2), and were higher than in control patients in 92–95% of the PDR cases, in line with previous studies^17,18,37–39^. Five other proteins showed elevated levels in PDR: MMP9 (48-fold), IL-8 (30-fold), adiponectin (18-fold), thrombospondin-1 (14-fold) and angiopoietin-2 (13-fold). These proteins may be important, because they could represent new targets for therapy.

MMP9 is a matrix metalloprotease that is activated via proteolytic cleavage by extracellular proteinases. When active it cleaves extracellular matrix proteins such as collagen IV, and it has been shown to be involved in leukocyte migration and blood-retinal barrier breakdown^40^. In the present study we show that MMP9 may also be related with increased fibrosis in PDR. In addition, MMP9, IGFBP3 and PDGF-BB levels were strongly related to the degree of vitreous haemorrhage (Table 4). IGFBP3 and PDGF-BB levels were also strongly related to visual acuity (Table3), which may relate to the reduced acuity associated with vitreous haemorrhage, rather than other causes. MMP9 is induced by conditions of inflammation, hypoxia or oxidative stress^41^ and it is also a pro-angiogenic protein that showed a strong correlation with VEGF levels in vitreous of PDR patients^40^. Between both proteins a positive feed-back loop of reciprocal induction in retinal pigment epithelial cells has been reported^42^. Although the molecular mechanisms of MMP9 have been studied primarily in vitro and remain to be confirmed in vivo, a variety of anti-MMP9 therapies have been suggested for the treatment of PDR^41^.

IL-8 is a major mediator of the inflammatory response by serving as a chemotactic factor and guiding the neutrophils to the site of infection, and is also a potent angiogenic factor. Elevated levels of IL-8 have been reported in DR and may have a role in inflammation and angiogenesis in DR^43^. IL-8 is, like VEGF, upregulated by hypoxia, but a direct correlation between both proteins in vitreous of PDR patients has not been reported. Interestingly, although PDR and DME are considered two different conditions with their own pathophysiology, the combination of elevated IL-8 and MCP1 was suggested as a marker of non-responsiveness to RZB therapy in DME^44^.

Adiponectin regulates lipid/glucose metabolism and anti-inflammatory and anti-angiogenic effects. Upregulated levels of adiponectin can be regarded as a compensatory and beneficial response to these effects and increasing evidence supports that it improves insulin-resistance and supports vascular maintenance in diabetic patients^45^.

For thrombospondin-1 (THBS1), which has anti-angiogenic effects, we could more accurately reproduce the findings of our previous study^34^, since protein levels were now within detectable range. Undetectable or decreased levels of THBS1 have been reported by others in vitreous from PDR patients^46,47^. It is detected as an abundant protein on platelets and is secreted by endothelial cells, fibroblasts, smooth muscle cells, and many other cells of the retina, including glial cells^48^. THBS1 exerts its anti-angiogenic effects through the CD36 receptor and its upregulation during PDR could thus be a compensatory mechanism against the pro-angiogenic actions of VEGF and other molecules.

Elevated levels of angiopoietin-2 in PDR have also been reported previously and pharmacological therapies with anti-ANG2 agents are currently being introduced for the treatment of DME^49^. ANG2 is also a pro-angiogenic factor, and its levels in vitreous of PDR patients were found to correlate with the levels of MMP9 and VEGF^50^. ANG2 has been implicated in DR as a co-mediator of blood-retinal barrier breakdown and in the detachment and migration of pericytes in preclinical DR^51^. In addition, it is involved as a co-factor in increased permeability and angiogenesis induced by VEGF in experimental models. The direct interaction of angiopoietin-2 with VEGF leads to destabilisation of the retinal vasculature^49^. In addition, cross-talk with VEGF-receptors, after binding of angiopoietin-2 to its receptor Tie2 may lead to destabilisation of the retinal vasculature as well^49^. In clinical trials in DME and nAMD, blocking of angiopoietin-2 in combination with VEGF inhibition by faricimab has shown comparable visual gains versus aflibercept in terms of efficacy, but possibly longer durability^52,53^, although results of these clinical studies should be interpreted with caution due to differences in the treatment regimens compared.

Our concept that proteins work in networks rather than as individual agents provides new insights to interpret our data. A strong correlation between the levels of two proteins in the individual samples, such as we observed for VEGF and PlGF, may suggest co-regulation at the pre- or post-transcriptional level. For VEGF and PlGF this is not surprising, as both proteins have synergistic effects, are regulated by hypoxia, and were found to play an important role in angiogenesis, as well as in the development of PDR^12^. In addition, of the quartet VEGF-PlGF-MCP1-ANG2, all except ANG2 were found to correlate with neovascularisation activity (Table 4). The central position of ANG2 that we found in this network of proteins is most interesting (Fig. 1). However, it does not necessarily imply that ANG2 plays a causative role in PDR as this could also be a secondary effect. Nevertheless, it is clear that there is co-regulation of ANG2 with many other proteins involved in PDR. This supports the previous notion that ANG2 may have a regulatory role in multiple processes that are context and environment dependent^49^. For example, increased ANG2 protein levels lead to decreased matrix attachment and contact with supporting cells, therefore allowing access for pro-angiogenic factors such as VEGF. ANG2 in the presence of VEGF may lead to neovascularisation, whereas in the absence of VEGF vessel regression may occur^49^. In our previous study, ANG2 protein levels were strongly correlated with the degree of fibrosis, and were in particular increased in PDR patients with fibrovascular membranes^34^. In the present study we could not confirm a correlation between ANG2 and fibrosis. However, MMP9, of which the protein levels strongly correlated with that of ANG2, was found to correlate with the degree of fibrosis (Table 4). ANG2 may therefore regulate fibrosis in conjunction with MMP9. Thus, the actions of ANG2 are probably context dependent and may be differentially regulated by the relative abundance of other proteins.

Many of the patients that had active PDR received anti-VEGF therapy 2–5 days prior to vitrectomy. AFB, directed against both VEGF and PlGF, was associated with a reduction in both growth factors as compared to the untreated PDR group. Patients treated with BVZ or RZB, also showed lower levels of PlGF. This is unexplained and others did not observe this decrease after BVZ treatment^26^. One possible cause is an indirect effect via decreased VEGF levels, since VEGF is able to induce PlGF expression^54^. Another possible explanation may be cessation of leakage from circulation after anti-VEGF therapy due to reduced vascular permeability. However, PlGF is probably not elevated by spill-over from the circulation by BRB breakdown, since reported protein levels in people with type 2 diabetes are much lower than the levels that we observed in vitreous^55^. Vitreous VEGF levels are also not influenced by the reported very low levels of VEGF in the circulation^56,57^.

Other proteins also showed decreased levels in the PDR-AFB group as compared to the PDR-U group (VEGFR1, GDNF, IL-1β, IL-10, NRG1, TNFα, PDGFAA, MCP1 and betacellulin). Decreased protein levels of IL-1β, IL-10, MCP1 and TNFα after intravitreal AFB have also been found by other researchers^24,58,59^. The mechanisms underlying these findings remain unknown. GDNF and MCP1 showed decreased levels in the PDR-BVZ/RZB group as well, suggesting that these proteins are regulated by VEGF levels. This may be explained by findings by others that VEGF reciprocally induces GDNF^60^ and MCP1 expression^61^. A notable difference observed after anti-VEGF treatment was the 13-fold lower levels of soluble VEGFR1 in the PDR-A group compared to the PDR-U group. VEGFR1 is a receptor for VEGF as well as PlGF. Reduced levels of this receptor may potentially be explained by an indirect effect of reduced VEGF and PlGF levels, or by direct binding of AFB to VEGFR1 alone or to its complex with VEGF and/or PlGF.

Protein levels of four proteins (IL-6, IL-8, angiopoietin-1 and galectin-3) and ProMMP9 activity were higher in the PDR-AFB group. The increase in galectin-3 (1.5-fold) in the PDR-AFB group is unexpected, given that AFB can bind galectin-1^28,29^. In our study, the protein levels of galectin-1 were unchanged in the PDR-AFB group compared with the PDR-U group. This may be explained by the fact that galectin-1 may be able to bind to AFB, but has a much lower binding affinity (K_D_ = 23.68 nM)^28^ than that of VEGF (K_D_ = 0.49 pM)^62^, meaning AFB preferentially binds to VEGF.

We acknowledge that our study has some limitations. We analysed a selection of proteins, and although we think we included most key proteins, we may have missed important proteins. The study also lacks data on the presence of macular oedema, as the majority of patients were treated for dense vitreous haemorrhages, making preoperative macula evaluation unreliable. Protein profiles may be different in patients with or without oedema. In addition, the selection of patients for anti-VEGF treatment were based on the severity of clinical manifestations, the criteria for which are described in detail in the “Methods” section. However, a strength of our study is that there was no selection bias confounding both anti-VEGF treatment groups, as the choice of anti-VEGF treatment was based solely on the local and temporary availability at the time, and no significant differences were found for clinical or demographic characteristics between these groups. To validate whether upregulation or downregulation of certain proteins after Aflibercept is a consistent response to the treatment would require additional prospective and ideally randomised studies.

In conclusion, we show a comprehensive analysis of the protein concentrations in vitreous of PDR patients treated with vitrectomy with or without VEGF pre-treatment, and patients with macular hole as controls. Our study confirms previous publications by ourselves and others, but also reveals many new insights. In the untreated PDR patients, we show that there are clusters of proteins that appear to be correlated. VEGF, PlGF, MCP1, but especially ANG2 appear to play a central role in this network of proteins. This suggests that ANG2 may have a regulatory function in the development of PDR, which is probably dependent on context and environmental factors. Pre-operative anti-VEGF treatment resulted in significantly higher or lower protein levels of a number of proteins depending on the use of AFB or BVZ/RBZ. These differentially affected protein networks after treatment of DR patients may explain the favourable short-term effects of AFB treatment compared with BVZ.

## Materials and methods

### Study population

The study followed the tenets of the Declaration of Helsinki, with approval from the National Health Service research ethics committee (South East Coast—Surrey research ethics committee reference 12/LO/0130). Informed consent was obtained from the subjects after explanation of the nature of the study. Consecutive patients undergoing vitrectomy for the complications of proliferative diabetic retinopathy by one surgeon from March 2015 to October 2018 were recruited (*n* = 112). The procedures for isolation and storage of vitreous samples was unchanged during this period. Eyes with previous vitrectomy surgery were excluded. Only one eye per patient was included. Eyes with active proliferative retinopathy or those requiring extensive dissection, as judged by the treating surgeon, were pre-treated with either intravitreal BVZ (*n* = 10), RZB (*n* = 3) (both Genentech, San Francisco, CA) or AFB (n = 25) (Regeneron, Tarrytown, NY), administered 2–5 days prior to vitrectomy based on the surgeon’s standard practice. There was no potential selection bias in the choice of these anti-VEGF agents, as they were used in consecutive time periods based on local and temporal availability of the drugs: BVZ was used in 2015, RZB in 2016 and AFB from 2017 to 2018. Active PDR requiring preoperative anti-VEGF administration was defined by a summation of clinical signs including the presence of proliferating perfused new vessels with budding tips or iris rubeosis, the presence of venous beading and/or widespread intraretinal haemorrhage and the absence of adequate previous pan retinal photocoagulation. No patients had anti-VEGF for DME less than 4 months preoperatively other than the dose given for surgery. A control group of non-diabetic patients undergoing surgery for idiopathic macular holes were also recruited and age and gender recorded (*n* = 52).

For PDR patients, a range of pre- and intraoperative variables were graded and recorded including age, gender, duration and type of diabetes, and glycosylated haemoglobin (HbA1c) level. The indication for surgery (namely either vitreous haemorrhage (VH), macular traction or traction retinal detachment (MT/TRD), combined tractional and rhegmatogenous retinal detachment (CTRD)) and the duration and density of any associated VH were recorded. The degree of VH was graded as 0 when there was no haemorrhage, as 1 when there was some hemorrhage but the fundal details could still be visualised on indirect ophthalmoscopy, as 2 when the optic disc was obscured by haemorrhage, and as 3 when no fundus details could be observed. The immediate preoperative visual acuity, and the presence of iris rubeosis were also recorded.

Fibrosis was graded as 0 when there was no fibrosis, as 1 when there were a few pre-retinal membranes (limited as in macular pucker), as 2 when white preretinal fibrotic membranes with limited extension into the vitreous cavity were present, and as 3 when abundant white membranes reaching into the vitreous body were observed. Neovascularization was graded as 0 when absent, as 1 (quiescent) when only non-perfused vessels were present, and as 2 (active) when there were perfused preretinal capillaries. Fibrovascular new vessel complexes were graded intraoperatively by their extent in disc areas as previously^63^ and the position of neovascularisation was recorded as none, disc attachment only, posterior pole attachment only or 1–4 quadrants of anterior vitreoretinal attachment^64^. Patients were followed up at 1 day, 2 weeks, and at 3 months postoperatively as a minimum. Visual acuity at 3 months and the occurrence of significant postoperative vitreous cavity haemorrhage (POVCH) requiring revision surgery, or revision vitrectomy for other indications was recorded.

### Sample collection

At the beginning of surgery prior to turning on the infusion, a ~ 0.5–1 ml undiluted mid-vitreous sample was obtained using a vitrectomy probe with a cut rate of 5000 cpm. The sample was immediately stored at − 80 °C. All surgeries were carried out by one surgeon using the Alcon Constellation 25G cannula (Alcon, Fort Worth, Texas, USA) with wide angle non-contact viewing using a standardised technique.

### Determination of protein levels

Vitreous samples were thawed and centrifuged for 15 min at 14,000 g at 4 °C to remove any cellular debris and supernatant was divided in aliquots of 50 µl and stored at − 80 °C until analysis. Proteins were first quantified by using a customizable array-based multiplex immunoassay (Human Quantibody array, RayBiotech, Norcross, GA, USA) and/or by sandwich enzyme-linked immunosorbent assay (ELISA). From each vitreous sample 200 µl was used per array. On a separate array, tenfold diluted samples were used for 3 proteins that were found to be abundantly expressed in our previous study (adiponectin, hepatocyte growth factor (HGF) and tissue inhibitor of metalloprotease-1 (TIMP1)) (Klaassen et al., 2017) for more accurate quantification. To confirm the Quantibody array results for key proteins, commercially available ELISA assays were used for VEGFA (Quantikine DVE00), PlGF (Quantikine DPG00) (both R&D Systems, Minneapolis MS, USA), pigment epithelium-derived factor (PEDF; ELH-SerpinF1) and insulin-like growth factor-binding protein 3 (IGFBP3; ELH-IGFBP3) (both RayBiotech), according to the manufacturer’s protocols. In addition, concentrations of connective tissue growth factor (CTGF) were determined by sandwich ELISA, using a modified protocol as described previously^65^. Two distinct monoclonal antibodies, specifically recognizing the N-terminal part of the CTGF protein, anti-CTGF module 1 (30D2) and module 2 (2–3) antibodies (Wako Pure Chemical Ind., Ltd., Osaka, Japan) were used for detection. With this method we detect the N-terminal part as well as the full length CTGF. Unfortunately, detection of only full-length CTGF with module 1 and module 4 antibodies did not work and we could therefore not subtract full-length CTGF to calculate the concentration of N-terminal CTGF^65^. The module 2 antibodies were labelled with biotin and desalted using the EZ-Link Sulfo-NHS-LC-Biotinylation kit (Cat#21435, Thermo Scientific) according to manufacturer's instructions. A 96-well plate was coated with anti-CTGF module 1 antibodies (5 µg/ml in PBS; 50 µl per well) and incubated at 4 °C overnight. Wells were washed 4 times with 300 µl PBS with 0.05% Tween20 (PBST). Blocking was performed by using PBST containing 1% bovine serum albumin (PBST-BSA, 100 µl per well) for 1 h at room temperature. Next, 50 µl standard (2 µg–3 ng/ml) and vitreous samples (diluted 2.5 times in PBST-BSA) were added and incubated for 1 h at room temperature. Purified recombinant human N-terminal CTGF fragment (LS-G11250, LSBio, Seattle, WA, USA) was used as standard. Wells were washed 4 times with PBST and incubated with biotin-labeled module 2 antibody (50 µl/well of 1 µg/ml in PBST-BSA) for 1 h at room temperature. After 4 times washing with PBST, 50 µL/well of HRP-streptavidin (Cat#M2032, Sanquin Reagents, Amsterdam, The Netherlands), diluted to 1 µg/mL with BSA-PBST was added and incubated for 30 min at room temperature. After 4 times washing with PBST, a colour reaction was achieved by adding 100 µL/well of TMB (3,3',5,5'-tetramethylbenzidine) reaction buffer (For one plate: 10 ml distilled water, 1.1 ml 1.1 M sodium-acetate, 110 µl TMB of 10 mg/ml, 1.1 µl 30% H_2_O_2_) and incubation for exactly 10 min at room temperature. The reaction was stopped by adding 50 µl of 1.5 N sulfuric acid and the plate was immediately read at 450 nm in a microplate reader (BioRad).

### Gelatin zymography

Activity of matrix metalloproteinases MMP2 and MMP9 was determined using a gelatin-zymography kit (Primary Cell, Hokkaido, Japan). Samples were randomly assigned to 15 gels, each gel containing 11 samples. Of all samples, 10 μl vitreous sample mixed with 10 μl loading buffer and 10 μl MMP marker were loaded onto a precast gel and processed according to the manufacturer’s directions. Bands on the zymograms were quantified using ImageJ software (https://imagej.nih.gov/ij/).

### Statistical analysis

Values of vitreous proteins are reported as a mean (pg/ml) ± standard deviation. Univariate analysis with two-tailed T-tests assuming unequal variance were performed to identify individual proteins significantly associated with PDR. A Bonferroni corrected *P* value of < 0.001 was considered to indicate statistically significant differences between protein levels on arrays and a *P* value < 0.01 for ELISAs; a *P* value < 0.01 was used to indicate statistically significant differences between clinical features. To compare array data with ELISA data, Bland and Altman plots were applied^33^. With this method the difference and mean of log10-values are plotted against each other and the slope of the regression line determined. If no significant change is observed, both methods are comparable. ANOVA or Kruskal–Wallis tests were used to analyse relations between protein concentrations and clinical features. Spearman’s rank correlation analysis (determined by SPSS, version 26) was used to investigate significant correlations between protein concentrations and clinical features or other proteins. Moderate correlations were defined as coefficients between 0.40 and 0.59 and strong correlations above 0.60.

## Supplementary Information

Supplementary Information 1.

Supplementary Information 2.

Supplementary Information 3.

Supplementary Information 4.

Supplementary Information 5.

## Data Availability

The data presented in this study are openly available in interactive form via figlinq.com (https://create.figlinq.com/dashboard/iklaassen:107).
