# Supplementary figures and images for: Vitreous protein networks around ANG2 and VEGF in proliferative diabetic retinopathy and the differential effects of aflibercept versus bevacizumab pre-treatment
Source: Sci Rep. 2022 Dec 6;12:21062. doi: 10.1038/s41598-022-25216-z (PMC9726866; doi:10.1038/s41598-022-25216-z)

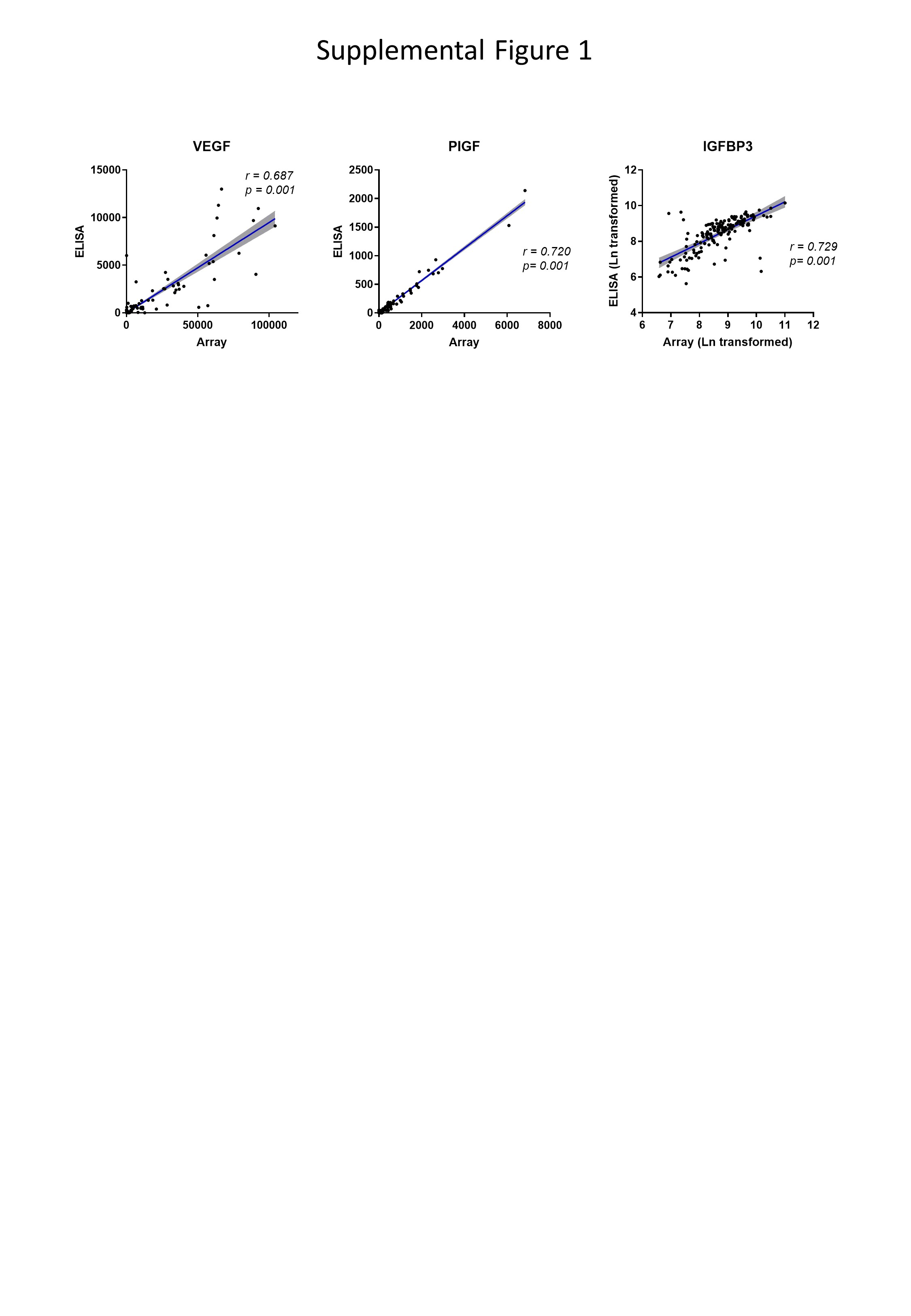

Supplement: Supplementary file 1 — Supplementary Information 1. [file 41598_2022_25216_MOESM1_ESM.jpg]

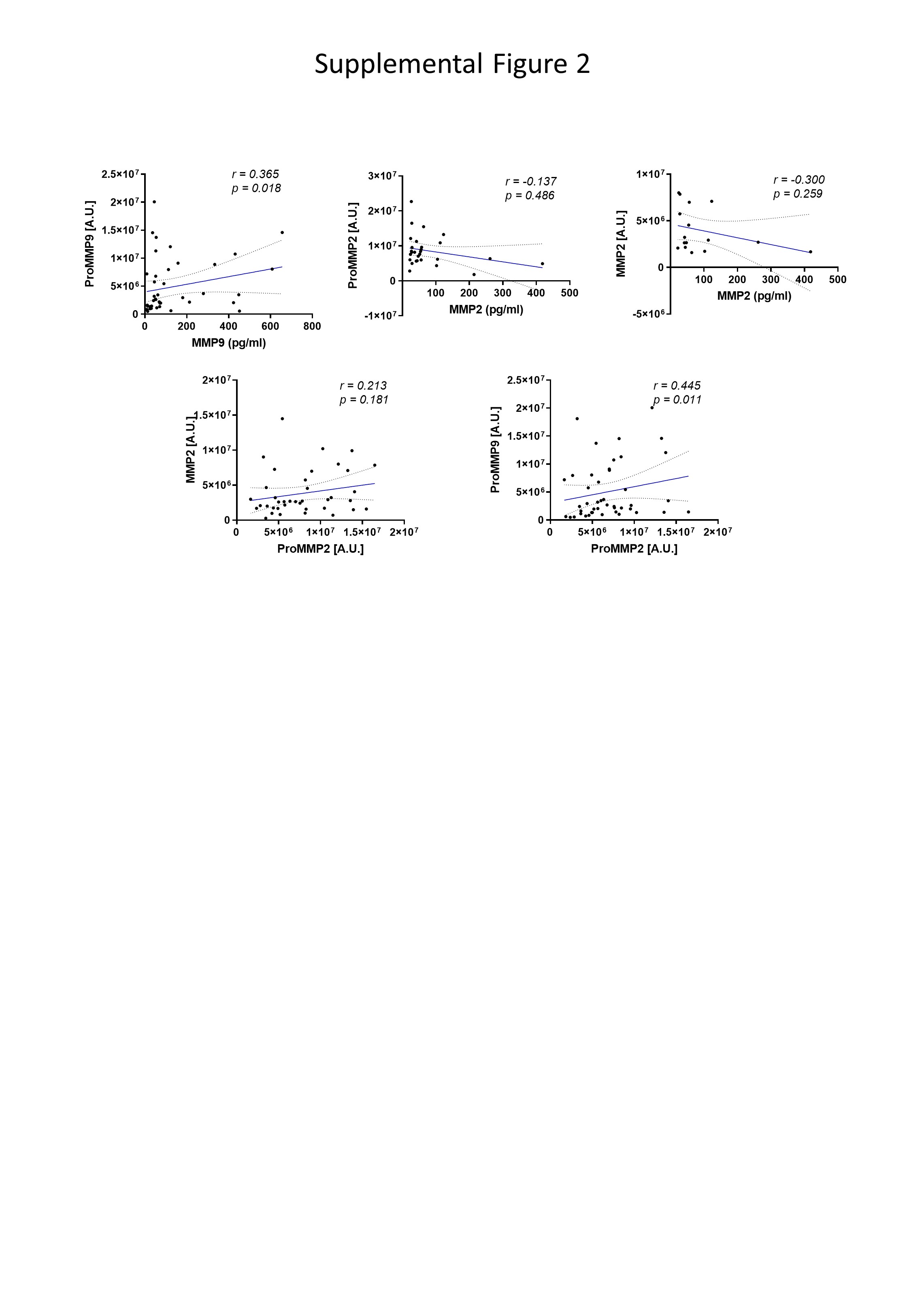

Supplement: Supplementary file 2 — Supplementary Information 2. [file 41598_2022_25216_MOESM2_ESM.jpg]
