# Supplementary material for: Vitreous protein networks around ANG2 and VEGF in proliferative diabetic retinopathy and the differential effects of aflibercept versus bevacizumab pre-treatment
Source: Sci Rep. 2022 Dec 6;12:21062. doi: 10.1038/s41598-022-25216-z (PMC9726866; doi:10.1038/s41598-022-25216-z)
Supplement: Supplementary file 3 — Supplementary Information 3. [file 41598_2022_25216_MOESM3_ESM.pdf]

**Supplemental Table 1**. Modelling of protein levels with group and gender as factors and age as a covariate.

| Variable | Control v PDR | | Age | | Gender | |
|---|---|---|---|---|---|---|
| All log transformed | *p* | Partial $R^2$, % | *p* | Partial $R^2$, % | *p* | Partial $R^2$, % |
| IGFBP3 ELISA | **<0.001** | 35.6 | 0.230 | 0.8 | 0.714 | 0.1 |
| PEDF ELISA | **<0.001** | 24.2 | 0.154 | 1.3 | 0.173 | 1.2 |
| Galectin 1 | **<0.001** | 45.2 | 0.013 | 2.7 | 0.321 | 0.4 |
| Galectin 3 | **<0.001** | 45.6 | 0.019 | 2.4 | 0.591 | 0.1 |
| GDF15 | **<0.001** | 50.9 | 0.003 | 3.5 | 0.434 | 0.2 |
| HGF | **<0.001** | 23.3 | 0.890 | 0.01 | 0.533 | 0.2 |
| ICAM1 | **<0.001** | 57.6 | 0.895 | <0.01 | 0.771 | 0.03 |
| IGFBP1 | **<0.001** | 35.6 | 0.294 | 0.6 | 0.757 | 0.05 |
| IGFBP3 | **<0.001** | 44.3 | 0.105 | 1.2 | 0.747 | <0.01 |
| MCP1 | **<0.001** | 50.3 | 0.536 | 0.2 | 0.954 | <0.01 |
| MMP2 | 0.022 | 4.2 | 0.654 | 0.2 | 0.508 | 0.3 |
| NOV | 0.007 | 5.7 | 0.110 | 1.9 | 0.191 | 1.3 |
| TGFb2 | 0.381 | 0.6 | 0.025 | 4.0 | 0.600 | 0.2 |
| VEGFR1 | **<0.001** | 24.8 | 0.620 | 0.2 | 0.969 | <0.01 |
| VEGFR2 | **<0.001** | 15.4 | 0.774 | 0.1 | 0.977 | <0.01 |
| VEGFA | **<0.001** | 65.4 | 0.002 | 2.5 | 0.371 | 0.2 |

Statistically significant differences with a P-value < 0.001 are indicated in bold.
