# Supplementary material for: Vitreous protein networks around ANG2 and VEGF in proliferative diabetic retinopathy and the differential effects of aflibercept versus bevacizumab pre-treatment
Source: Sci Rep. 2022 Dec 6;12:21062. doi: 10.1038/s41598-022-25216-z (PMC9726866; doi:10.1038/s41598-022-25216-z)
Supplement: Supplementary file 4 — Supplementary Information 4. [file 41598_2022_25216_MOESM4_ESM.pdf]

Supplemental Table 2. Spearman's rank correlation between protein concentrations in vitreous of untreated PDR patients.

		ADIPOQ	ANGPT1	ANGPT2	BTC	CTGF	LGALS1	LGALS3	GDF15	GDNF	HGF	ICAM1	IGFBP1	IGFBP3	IL-1β	IL-6
**ADIPOQ**	R	1.000	0.194	,344**	0.029	,361**	,308**	,264*	,392**	0.068	,332**	,433**	,478**	,549**	0.189	,268*
	P		0.140	0.003	0.923	0.002	0.008	0.024	0.001	0.757	0.004	0.000	0.000	0.000	0.468	0.021
	N	74	59	74	14	71	74	73	74	23	74	74	60	74	17	74
**ANGPT1**	R	0.194	1.000	-0.044	-0.420	,263*	-0.033	-0.030	0.113	0.240	0.112	0.146	-0.058	0.252	-0.242	0.240
	P	0.140		0.742	0.175	0.050	0.806	0.820	0.392	0.322	0.400	0.269	0.696	0.054	0.426	0.067
	N	59	59	59	12	56	59	58	59	19	59	59	48	59	13	59
**ANGPT2**	R	,344**	-0.044	1.000	-0.095	,478**	,624**	,252*	,480**	-0.233	,477**	,544**	,265*	,518**	-0.184	,439**
	P	0.003	0.742		0.748	0.000	0.000	0.031	0.000	0.284	0.000	0.000	0.041	0.000	0.480	0.000
	N	74	59	74	14	71	74	73	74	23	74	74	60	74	17	74
**BTC**	R	0.029	-0.420	-0.095	1.000	0.130	-0.090	-0.169	-0.385	,636*	-0.064	-0.429	0.280	-0.354	0.086	-0.055
	P	0.923	0.175	0.748		0.659	0.759	0.563	0.175	0.048	0.829	0.126	0.379	0.215	0.872	0.852
	N	14	12	14	14	14	14	14	14	10	14	14	12	14	6	14
**CTGF**	R	,361**	,263*	,478**	0.130	1.000	,280*	0.200	,552**	0.001	,452**	,372**	,350**	,547**	-0.243	,361**
	P	0.002	0.050	0.000	0.659		0.018	0.097	0.000	0.998	0.000	0.001	0.007	0.000	0.348	0.002
	N	71	56	71	14	73	71	70	71	22	71	71	58	71	17	71
**LGALS1**	R	,308**	-0.033	,624**	-0.090	,280*	1.000	,513**	,547**	0.149	,297*	,706**	,336**	,660**	0.105	,352**
	P	0.008	0.806	0.000	0.759	0.018		0.000	0.000	0.497	0.010	0.000	0.009	0.000	0.687	0.002
	N	74	59	74	14	71	74	73	74	23	74	74	60	74	17	74
**LGALS3**	R	,264*	-0.030	,252*	-0.169	0.200	,513**	1.000	,325**	-0.030	0.041	,520**	0.143	,424**	,510*	0.070
	P	0.024	0.820	0.031	0.563	0.097	0.000		0.005	0.893	0.732	0.000	0.279	0.000	0.037	0.558
	N	73	58	73	14	70	73	73	73	23	73	73	59	73	17	73
**GDF15**	R	,392**	0.113	,480**	-0.385	,552**	,547**	,325**	1.000	-0.219	,417**	,600**	,413**	,658**	0.179	,417**
	P	0.001	0.392	0.000	0.175	0.000	0.000	0.005		0.315	0.000	0.000	0.001	0.000	0.492	0.000
	N	74	59	74	14	71	74	73	74	23	74	74	60	74	17	74
**GDNF**	R	0.068	0.240	-0.233	,636*	0.001	0.149	-0.030	-0.219	1.000	0.228	-0.145	-0.097	-0.102	0.286	-0.131
	P	0.757	0.322	0.284	0.048	0.998	0.497	0.893	0.315		0.295	0.508	0.674	0.644	0.493	0.550
	N	23	19	23	10	22	23	23	23	23	23	23	21	23	8	23
**HGF**	R	,332**	0.112	,477**	-0.064	,452**	,297*	0.041	,417**	0.228	1.000	,443**	,366**	,379**	-0.221	,317**
	P	0.004	0.400	0.000	0.829	0.000	0.010	0.732	0.000	0.295		0.000	0.004	0.001	0.395	0.006
	N	74	59	74	14	71	74	73	74	23	74	74	60	74	17	74
**ICAM1**	R	,433**	0.146	,544**	-0.429	,372**	,706**	,520**	,600**	-0.145	,443**	1.000	,403**	,599**	0.076	,485**
	P	0.000	0.269	0.000	0.126	0.001	0.000	0.000	0.000	0.508	0.000		0.001	0.000	0.772	0.000
	N	74	59	74	14	71	74	73	74	23	74	74	60	74	17	74
**IGFBP1**	R	,478**	-0.058	,265*	0.280	,350**	,336**	0.143	,413**	-0.097	,366**	,403**	1.000	,467**	0.429	,260*
	P	0.000	0.696	0.041	0.379	0.007	0.009	0.279	0.001	0.674	0.004	0.001		0.000	0.126	0.045
	N	60	48	60	12	58	60	59	60	21	60	60	60	60	14	60
**IGFBP3**	R	,549**	0.252	,518**	-0.354	,547**	,660**	,424**	,658**	-0.102	,379**	,599**	,467**	1.000	0.022	,379**
	P	0.000	0.054	0.000	0.215	0.000	0.000	0.000	0.000	0.644	0.001	0.000	0.000		0.933	0.001
	N	74	59	74	14	71	74	73	74	23	74	74	60	74	17	74
**IL-1β**	R	0.189	-0.242	-0.184	0.086	-0.243	0.105	,510*	0.179	0.286	-0.221	0.076	0.429	0.022	1.000	-0.130
	P	0.468	0.426	0.480	0.872	0.348	0.687	0.037	0.492	0.493	0.395	0.772	0.126	0.933		0.619
	N	17	13	17	6	17	17	17	17	8	17	17	14	17	17	17
**IL-6**	R	,268*	0.240	,439**	-0.055	,361**	,352**	0.070	,417**	-0.131	,317**	,485**	,260*	,379**	-0.130	1.000
	P	0.021	0.067	0.000	0.852	0.002	0.002	0.558	0.000	0.550	0.006	0.000	0.045	0.001	0.619	
	N	74	59	74	14	71	74	73	74	23	74	74	60	74	17	74
**IL-8**	R	0.220	0.080	,473**	-0.503	,446**	,401**	-0.007	,428**	-0.089	,435**	,399**	0.221	,499**	-0.424	,449**
	P	0.059	0.545	0.000	0.067	0.000	0.000	0.952	0.000	0.687	0.000	0.000	0.090	0.000	0.090	0.000
	N	74	59	74	14	71	74	73	74	23	74	74	60	74	17	74
**MCP1**	R	0.181	-0.132	,614**	-0.152	,282*	,359**	0.027	,387**	0.037	,392**	0.205	0.097	,321**	-0.113	,266*
	P	0.123	0.318	0.000	0.605	0.017	0.002	0.818	0.001	0.868	0.001	0.080	0.462	0.005	0.667	0.022
	N	74	59	74	14	71	74	73	74	23	74	74	60	74	17	74
**MMP2**	R	,426*	0.216	,777**	-0.657	0.362	,573**	,505**	,433*	-0.224	,435*	,469**	0.107	,624**	-0.167	0.303
	P	0.017	0.260	0.000	0.156	0.054	0.001	0.004	0.015	0.484	0.014	0.008	0.611	0.000	0.668	0.097
	N	31	29	31	6	29	31	30	31	12	31	31	25	31	9	31
**MMP9**	R	0.046	0.039	,653**	-0.336	,354**	,324**	0.178	,336**	-0.045	,349**	,322*	0.080	,288*	-0.260	,327**
	P	0.718	0.789	0.000	0.240	0.005	0.010	0.162	0.007	0.844	0.005	0.010	0.571	0.022	0.314	0.009
	N	63	50	63	14	61	63	63	63	22	63	63	53	63	17	63
**NOV**	R	0.186	-0.106	,365**	-0.160	,369**	0.184	-0.090	,302**	-0.059	,294*	0.081	0.071	0.206	-0.275	0.120
	P	0.113	0.425	0.001	0.584	0.002	0.117	0.451	0.009	0.788	0.011	0.495	0.591	0.078	0.286	0.309
	N	74	59	74	14	71	74	73	74	23	74	74	60	74	17	74
**PDGFAA**	R	0.186	-0.005	,465**	-0.020	,263*	,583**	,506**	,335**	0.091	,272*	,588**	,283*	,445**	0.248	,262*
	P	0.129	0.971	0.000	0.946	0.035	0.000	0.000	0.005	0.680	0.025	0.000	0.033	0.000	0.338	0.031
	N	68	53	68	14	65	68	67	68	23	68	68	57	68	17	68
**PEDF**	R	-0.095	,541**	0.039	-0.126	0.166	-0.042	-,253*	0.097	0.253	,301*	-0.027	-0.018	0.009	-0.447	,318**
	P	0.446	0.000	0.756	0.681	0.174	0.734	0.040	0.435	0.283	0.013	0.826	0.898	0.946	0.083	0.009
	N	67	54	67	13	69	67	66	67	20	67	67	54	67	16	67
**PIGF**	R	,331**	-0.235	,712**	0.297	,362**	,426**	0.180	,347**	-0.043	,231*	,319**	0.081	,303**	0.044	,399**
	P	0.004	0.075	0.000	0.303	0.002	0.000	0.130	0.003	0.844	0.049	0.006	0.540	0.009	0.866	0.000
	N	73	58	73	14	70	73	72	73	23	73	73	59	73	17	73
**TGFβ2**	R	-0.168	,346**	0.004	-0.253	0.188	-0.057	-,331**	0.116	-0.074	0.113	-0.055	0.083	0.159	-0.253	0.218
	P	0.164	0.008	0.974	0.405	0.128	0.637	0.005	0.339	0.750	0.350	0.653	0.543	0.188	0.345	0.070
	N	70	57	70	13	67	70	69	70	21	70	70	56	70	16	70
**THBS1**	R	0.166	,333*	,304*	-0.174	,480**	,306*	,261*	,365**	-0.023	0.220	,331**	-0.003	,426**	-0.027	0.149
	P	0.175	0.013	0.012	0.553	0.000	0.011	0.033	0.002	0.919	0.072	0.006	0.983	0.000	0.918	0.225
	N	68	55	68	14	65	68	67	68	22	68	68	58	68	17	68
**TIMP1**	R	0.013	-0.083	,248*	0.024	0.194	-0.024	0.149	0.053	-0.022	0.209	0.128	-0.023	0.067	-0.015	-0.008
	P	0.910	0.530	0.033	0.935	0.104	0.840	0.209	0.656	0.922	0.073	0.279	0.860	0.570	0.955	0.948
	N	74	59	74	14	71	74	73	74	23	74	74	60	74	17	74
**UBB+1**	R	0.367	0.247	0.014	0.700	-0.100	0.119	0.075	-0.005	0.500	0.188	0.114	-0.044	0.059	,821*	0.075
	P	0.112	0.415	0.955	0.188	0.684	0.618	0.753	0.985	0.170	0.427	0.631	0.871	0.806	0.023	0.753
	N	20	13	20	5	19	20	20	20	9	20	20	16	20	7	20
**VEGFR1**	R	0.216	0.095	,348**	-0.385	,327**	,343**	0.116	,377**	0.126	,612**	,505**	,436**	,358**	-0.130	,398**
	P	0.065	0.473	0.002	0.175	0.005	0.003	0.328	0.001	0.565	0.000	0.000	0.000	0.002	0.619	0.000
	N	74	59	74	14	71	74	73	74	23	74	74	60	74	17	74
**VEGFR2**	R	,366**	0.145	,452**	-0.266	,239*	,452**	,400**	,453**	-0.223	,335**	,680**	,255*	,398**	0.191	,570**
	P	0.001	0.273	0.000	0.358	0.045	0.000	0.000	0.000	0.306	0.004	0.000	0.050	0.000	0.462	0.000
	N	74	59	74	14	71	74	73	74	23	74	74	60	74	17	74
**VEGFA**	R	,271*	-0.207	,640**	-0.068	,327**	,354**	0.212	,328**	-0.045	,269*	0.211	0.057	,375**	0.049	0.130
	P	0.022	0.126	0.000	0.817	0.007	0.002	0.079	0.005	0.837	0.023	0.077	0.673	0.001	0.852	0.280
	N	71	56	71	14	68	71	70	71	23	71	71	58	71	17	71

		IL-8	MCP1	MMP2	MMP9	NOV	PDGFAA	PEDF	PIGF	TGFβ2	THSB1	TIMP1	UBB+1	VEGFR1	VEGFR2	VEGFA
**ADIPOQ**	R	0.220	0.181	,426*	0.046	0.186	0.186	-0.095	,331**	-0.168	0.166	0.013	0.367	0.216	,366**	,271*
	P	0.059	0.123	0.017	0.718	0.113	0.129	0.446	0.004	0.164	0.175	0.910	0.112	0.065	0.001	0.022
	N	74	74	31	63	74	68	67	73	70	68	74	20	74	74	71
**ANGPT1**	R	0.080	-0.132	0.216	0.039	-0.106	-0.005	,541**	-0.235	,346**	,333*	-0.083	0.247	0.095	0.145	-0.207
	P	0.545	0.318	0.260	0.789	0.425	0.971	0.000	0.075	0.008	0.013	0.530	0.415	0.473	0.273	0.126
	N	59	59	29	50	59	53	54	58	57	55	59	13	59	59	56
**ANGPT2**	R	,473**	,614**	,777**	,653**	,365**	,465**	0.039	,712**	0.004	,304*	,248*	0.014	,348**	,452**	,640**
	P	0.000	0.000	0.000	0.000	0.001	0.000	0.756	0.000	0.974	0.012	0.033	0.955	0.002	0.000	0.000
	N	74	74	31	63	74	68	67	73	70	68	74	20	74	74	71
**BTC**	R	-0.503	-0.152	-0.657	-0.336	-0.160	-0.020	-0.126	0.297	-0.253	-0.174	0.024	0.700	-0.385	-0.266	-0.068
	P	0.067	0.605	0.156	0.240	0.584	0.946	0.681	0.303	0.405	0.553	0.935	0.188	0.175	0.358	0.817
	N	14	14	6	14	14	14	13	14	13	14	14	5	14	14	14
**CTGF**	R	,446**	,282*	0.362	,354**	,369**	,263*	0.166	,362**	0.188	,480**	0.194	-0.100	,327**	,239*	,327**
	P	0.000	0.017	0.054	0.005	0.002	0.035	0.174	0.002	0.128	0.000	0.104	0.684	0.005	0.045	0.007
	N	71	71	29	61	71	65	69	70	67	65	71	19	71	71	68
**LGALS1**	R	,401**	,359**	,573**	,324**	0.184	,583**	-0.042	,426**	-0.057	,306*	-0.024	0.119	,343**	,452**	,354**
	P	0.000	0.002	0.001	0.010	0.117	0.000	0.734	0.000	0.637	0.011	0.840	0.618	0.003	0.000	0.002
	N	74	74	31	63	74	68	67	73	70	68	74	20	74	74	71
**LGALS3**	R	-0.007	0.027	,505**	0.178	-0.090	,506**	-,253*	0.180	-,331**	,261*	0.149	0.075	0.116	,400**	0.212
	P	0.952	0.818	0.004	0.162	0.451	0.000	0.040	0.130	0.005	0.033	0.209	0.753	0.328	0.000	0.079
	N	73	73	30	63	73	67	66	72	69	67	73	20	73	73	70
**GDF15**	R	,428**	,387**	,433*	,336**	,302**	,335**	0.097	,347**	0.116	,365**	0.053	-0.005	,377**	,453**	,328**
	P	0.000	0.001	0.015	0.007	0.009	0.005	0.435	0.003	0.339	0.002	0.656	0.985	0.001	0.000	0.005
	N	74	74	31	63	74	68	67	73	70	68	74	20	74	74	71
**GDNF**	R	-0.089	0.037	-0.224	-0.045	-0.059	0.091	0.253	-0.043	-0.074	-0.023	-0.022	0.500	0.126	-0.223	-0.045
	P	0.687	0.868	0.484	0.844	0.788	0.680	0.283	0.844	0.750	0.919	0.922	0.170	0.565	0.306	0.837
	N	23	23	12	22	23	23	20	23	21	22	23	9	23	23	23
**HGF**	R	,435**	,392**	,435*	,349**	,294*	,272*	,301*	,231*	0.113	0.220	0.209	0.188	,612**	,335**	,269*
	P	0.000	0.001	0.014	0.005	0.011	0.025	0.013	0.049	0.350	0.072	0.073	0.427	0.000	0.004	0.023
	N	74	74	31	63	74	68	67	73	70	68	74	20	74	74	71
**ICAM1**	R	,399**	0.205	,469**	,322*	0.081	,588**	-0.027	,319**	-0.055	,331**	0.128	0.114	,505**	,680**	0.211
	P	0.000	0.080	0.008	0.010	0.495	0.000	0.826	0.006	0.653	0.006	0.279	0.631	0.000	0.000	0.077
	N	74	74	31	63	74	68	67	73	70	68	74	20	74	74	71
**IGFBP1**	R	0.221	0.097	0.107	0.080	0.071	,283*	-0.018	0.081	0.083	-0.003	-0.023	-0.044	,436**	,255*	0.057
	P	0.090	0.462	0.611	0.571	0.591	0.033	0.898	0.540	0.543	0.983	0.860	0.871	0.000	0.050	0.673
	N	60	60	25	53	60	57	54	59	56	58	60	16	60	60	58
**IGFBP3**	R	,499**	,321**	,624**	,288*	0.206	,445**	0.009	,303**	0.159	,426**	0.067	0.059	,358**	,398**	,375**
	P	0.000	0.005	0.000	0.022	0.078	0.000	0.946	0.009	0.188	0.000	0.570	0.806	0.002	0.000	0.001
	N	74	74	31	63	74	68	67	73	70	68	74	20	74	74	71
**IL-1β**	R	-0.424	-0.113	-0.167	-0.260	-0.275	0.248	-0.447	0.044	-0.253	-0.027	-0.015	,821*	-0.130	0.191	0.049
	P	0.090	0.667	0.668	0.314	0.286	0.338	0.083	0.866	0.345	0.918	0.955	0.023	0.619	0.462	0.852
	N	17	17	9	17	17	17	16	17	16	17	17	7	17	17	17
**IL-6**	R	,449**	,266*	0.303	,327**	0.120	,262*	,318**	,399**	0.218	0.149	-0.008	0.075	,398**	,570**	0.130
	P	0.000	0.022	0.097	0.009	0.309	0.031	0.009	0.000	0.070	0.225	0.948	0.753	0.000	0.000	0.280
	N	74	74	31	63	74	68	67	73	70	68	74	20	74	74	71
**IL-8**	R	1.000	,528**	,514**	,426**	0.228	,295*	0.204	,268*	0.164	,417**	0.127	-0.126	,408**	0.174	,298*
	P		0.000	0.003	0.000	0.050	0.015	0.098	0.022	0.174	0.000	0.282	0.596	0.000	0.139	0.012
	N	74	74	31	63	74	68	67	73	70	68	74	20	74	74	71
**MCP1**	R	,528**	1.000	0.329	,431**	,366**	,243*	0.130	,615**	0.176	0.099	0.049	0.075	,302**	0.143	,726**
	P	0.000		0.070	0.000	0.001	0.046	0.294	0.000	0.146	0.423	0.680	0.753	0.009	0.224	0.000
	N	74	74	31	63	74	68	67	73	70	68	74	20	74	74	71
**MMP2**	R	,514**	0.329	1.000	,398*	0.231	,539**	-0.100	0.324	-0.008	0.299	0.282	0.071	,360*	0.353	0.337
	P	0.003	0.070		0.040	0.211	0.002	0.604	0.075	0.966	0.103	0.125	0.867	0.047	0.051	0.064
	N	31	31	31	27	31	30	29	31	29	31	31	8	31	31	31
**MMP9**	R	,426**	,431**	,398*	1.000	0.069	0.219	0.116	,422**	0.024	,316*	0.163	0.261	,258*	0.216	,339**
	P	0.000	0.000	0.040		0.591	0.102	0.390	0.001	0.858	0.015	0.201	0.295	0.041	0.090	0.007
	N	63	63	27	63	63	57	57	62	60	59	63	18	63	63	62
**NOV**	R	0.228	,366**	0.231	0.069	1.000	0.102	0.097	,400**	0.216	,258*	0.031	-0.165	0.149	0.054	,397**
	P	0.050	0.001	0.211	0.591		0.406	0.436	0.000	0.072	0.034	0.791	0.486	0.205	0.651	0.001
	N	74	74	31	63	74	68	67	73	70	68	74	20	74	74	71
**PDGFAA**	R	,295*	,243*	,539**	0.219	0.102	1.000	-0.138	,290*	-0.051	,302*	,402**	-0.114	,292*	,361**	,364**
	P	0.015	0.046	0.002	0.102	0.406		0.290	0.016	0.689	0.015	0.001	0.631	0.016	0.002	0.003
	N	68	68	30	57	68	68	61	68	64	64	68	20	68	68	66
**PEDF**	R	0.204	0.130	-0.100	0.116	0.097	-0.138	1.000	0.014	,493**	0.164	-0.122	-0.044	0.202	-0.038	-0.013
	P	0.098	0.294	0.604	0.390	0.436	0.290		0.912	0.000	0.206	0.326	0.858	0.102	0.759	0.921
	N	67	67	29	57	67	61	69	66	63	61	67	19	67	67	64
**PIGF**	R	,268*	,615**	0.324	,422**	,400**	,290*	0.014	1.000	-0.014	0.155	0.137	-0.018	0.142	,303**	,840**
	P	0.022	0.000	0.075	0.001	0.000	0.016	0.912		0.912	0.206	0.247	0.940	0.229	0.009	0.000
	N	73	73	31	62	73	68	66	73	69	68	73	20	73	73	71
**TGFβ2**	R	0.164	0.176	-0.008	0.024	0.216	-0.051	,493**	-0.014	1.000	0.031	-0.220	-0.056	0.136	-0.052	0.019
	P	0.174	0.146	0.966	0.858	0.072	0.689	0.000	0.912		0.806	0.068	0.830	0.263	0.669	0.877
	N	70	70	29	60	70	64	63	69	70	64	70	17	70	70	67
**THBS1**	R	,417**	0.099	0.299	,316*	,258*	,302*	0.164	0.155	0.031	1.000	0.156	0.009	0.064	0.159	0.208
	P	0.000	0.423	0.103	0.015	0.034	0.015	0.206	0.206	0.806		0.205	0.972	0.606	0.196	0.092
	N	68	68	31	59	68	64	61	68	64	68	68	19	68	68	67
**TIMP1**	R	0.127	0.049	0.282	0.163	0.031	,402**	-0.122	0.137	-0.220	0.156	1.000	-0.295	0.058	-0.031	0.201
	P	0.282	0.680	0.125	0.201	0.791	0.001	0.326	0.247	0.068	0.205		0.207	0.623	0.791	0.092
	N	74	74	31	63	74	68	67	73	70	68	74	20	74	74	71
**UBB+1**	R	-0.126	0.075	0.071	0.261	-0.165	-0.114	-0.044	-0.018	-0.056	0.009	-0.295	1.000	-0.069	0.235	-0.191
	P	0.596	0.753	0.867	0.295	0.486	0.631	0.858	0.940	0.830	0.972	0.207		0.772	0.319	0.433
	N	20	20	8	18	20	20	19	20	17	19	20	20	20	20	19
**VEGFR1**	R	,408**	,302**	,360*	,258*	0.149	,292*	0.202	0.142	0.136	0.064	0.058	-0.069	1.000	,457**	0.147
	P	0.000	0.009	0.047	0.041	0.205	0.016	0.102	0.229	0.263	0.606	0.623	0.772		0.000	0.222
	N	74	74	31	63	74	68	67	73	70	68	74	20	74	74	71
**VEGFR2**	R	0.174	0.143	0.353	0.216	0.054	,361**	-0.038	,303**	-0.052	0.159	-0.031	0.235	,457**	1.000	0.176
	P	0.139	0.224	0.051	0.090	0.651	0.002	0.759	0.009	0.669	0.196	0.791	0.319	0.000		0.143
	N	74	74	31	63	74	68	67	73	70	68	74	20	74	74	71
**VEGFA**	R	,298*	,726**	0.337	,339**	,397**	,364**	-0.013	,840**	0.019	0.208	0.201	-0.191	0.147	0.176	1.000
	P	0.012	0.000	0.064	0.007	0.001	0.003	0.921	0.000	0.877	0.092	0.092	0.433	0.222	0.143	
	N	71	71	31	62	71	66	64	71	67	67	71	19	71	71	71

Spearman's rank correlation analysis showing regression coefficient (R), P-value (P) and number of included cases (N).

Strong and moderate correlations with a *P* -value <0.01 are indicated in yellow and blue, respectively.

*Correlation is significant at the 0.05 level (2-tailed).

**Correlation is significant at the 0.01 level (2-tailed).
