# Supplementary material for: Vitreous protein networks around ANG2 and VEGF in proliferative diabetic retinopathy and the differential effects of aflibercept versus bevacizumab pre-treatment
Source: Sci Rep. 2022 Dec 6;12:21062. doi: 10.1038/s41598-022-25216-z (PMC9726866; doi:10.1038/s41598-022-25216-z)
Supplement: Supplementary file 5 — Supplementary Information 5. [file 41598_2022_25216_MOESM5_ESM.docx]

**Suppl. Figure 1. Correlation plots of ELISA versus Array data for VEGF, PlGF and IGFBP3.** All data points of ELISA and array are plotted against each other for each protein. A best-fitted linear regression line with 95% confidence bands is shown. To correct for a skewed data distribution, the data points of IGFBP3 were natural-log transformed. Significant (*P* = 0.001) Pearson’s correlations (R) within all samples were found for each protein.

**Suppl. Figure 2. Correlation plots of Zymography data versus Array data.** All data points of zymography data and array are plotted against each other for each protein. A best-fitted linear regression line with 95% confidence bands is shown. Zymography data is expressed as intensity of bands in arbitrary units (A.U.), array data is expressed in pg/ml. Significant (*P* = 0.001) Pearson’s correlations (*r*) were found between MMP9 protein levels and ProMMP9 activity and between ProMMP2 activity and ProMMP9 activity.
